# High-performance membranes with full pH-stability†

**DOI:** 10.1039/c7ra13663c

**Published:** 2018-02-26

**Authors:** Nick Daems, Sam Milis, Rhea Verbeke, Anthony Szymczyk, Paolo P. Pescarmona, Ivo F. J. Vankelecom

**Affiliations:** Centre for Surface Chemistry and Catalysis, KU Leuven Celestijnenlaan 200F 3001 Heverlee Belgium ivo.vankelecom@kuleuven.be; Univ Rennes, CNRS, ISCR (Institut des Sciences Chimiques de Rennes) – UMR 6226 F-35000 Rennes France; Chemical Engineering Group, Engineering and Technology Institute Groningen (ENTEG), University of Groningen, Nijenborgh 4 9749 AG Groningen The Netherlands

## Abstract

Following current strong demands from, among others, paper, food and mining industries, a novel type of nanofiltration membrane was developed, which displays excellent performance in terms of selectivity/flux with a unique combination of chemical stability over the full (0–14) pH-range and thermal stability up to 120 °C. The membrane consists of polyvinylidene fluoride grafted with polystyrene sulfonic acid. The optimum membrane showed water permeances of 2.4 L h^−1^ m^−2^ bar^−1^ while retaining NaCl, MgSO_4_ and Rhodamine B (479 Da) for respectively ≈60%, ≈80% and >96%.

## Abstract 2

Grafted PVDF membranes were synthesized with improved nanofiltration properties (*R*_NaCl_ ≈ 60%) and stability over the full pH range.

## Graphical abstract

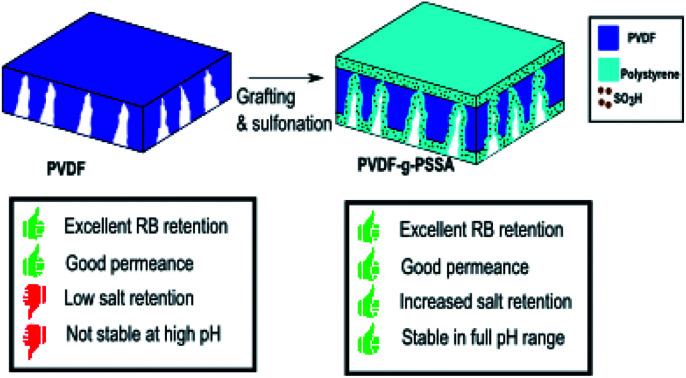

## 1. Introduction

Nanofiltration (NF) membranes currently attract a lot of interest, as they are able to separate rather small compounds (*e.g.* sugars, micropollutants, dyes or multi-valent ionic compounds) from water or other liquids. This offers an economical and environmentally benign alternative to conventional separation technologies by exploiting the general advantages of membrane technology, *i.e.* easy upscaling, no waste generation and low energy consumption.^1–5^ NF-membranes can be used in a broad range of areas, including water treatment, food processing and biotechnology.^5–7^

Most commercially available NF membranes that are applied in industrial environments are either amide or cellulose acetate based and have thus far shown limited stability at extreme pH conditions (above 12 or below 2) as they obviously suffer from hydrolysis under these conditions. Especially membranes that can withstand extreme alkaline conditions (pH 12 or higher) are scarce.^3,4,6^ In addition, most membranes completely lose their integrity when exposed to high temperatures (>100 °C). For many industrial applications which are either operated or which involve cleaning procedures under such aggressive conditions, the use of membranes is currently excluded. Therefore, developing membranes that are stable in a wider pH-window would largely expand the potential use of NF membranes and render many large-scale industrial processes more efficient from an economic and sustainability point of view.^2–4,7–9^ At low pH conditions, possible applications would include metal-ion removal from metallurgy process streams^7^ or sulfate-ion removal from streams originating from the mining industry.^10–12^ At high pH, filtration and reuse of cleaning solutions from the food and dairy industry, or the effluent treatment in pulp, paper and textile industry would benefit from the use of such NF membranes.^4,13–15^ In addition, even when temperature or pH-stability is not of prime importance during the actual filtration, it would allow implementation of more aggressive, hence more effective, cleaning procedures to fully recover the original membrane performance and extend membrane lifetimes.^16^ Even though the range of potential applications is thus broad, reports on pH-resistant NF membranes have been limited up until now, mainly owing to the difficulty of finding materials with intrinsic thermal, mechanical and chemical stability that can be turned into relatively dense membranes.

There are currently only few commercially available membranes that allow operation under extreme pH. NP030 from Microdyn Nadir and HYDRACoRe from Hydranautics/Nitto Denko, both consist of sulfonated polyethersulfone, while Duracid® from GE, MPF-34 from Koch Membrane systems and A3014 from Advanced Membrane System Technologies have undisclosed compositions. B4022 from Advanced Membrane System Technologies is a melamine–polyamine membrane that is stable at alkaline pH. Inopor® supplies ceramic membranes with a good stability (SiO_2_ or TiO_2_, both pH 0–14).^3,7,9,17^ However, all have their specific drawbacks, either due to a rather high molecular weight cut-off (≥500 g mol^−1^), narrow temperature application range (10–80 °C)^12^ or a low permeance (<1.5 L h^−1^ m^−2^ bar^−1^).^3,18,19^ All of the above drawbacks limit their commercialization and broad-scale application. The search for alternative pH-resistant NF membranes thus remains of high importance, as can be seen from the rising amount of publications in this research field over the past few years. Unfortunately, they are all limited to membranes with stability in acidic environment.^7,11,12,19^ On the other hand, alkaline-stable membranes are known in the field of anion-exchange membrane fuel cells. However, none of them have so far been tuned to also become applicable in NF.^20,21^ Thus, not a single NF membrane currently exists that is stable at both high and low pH.

Several of the commercial cation-exchange membranes consist of sulfonated aromatic polymers, which have the advantage of being intrinsically chemically much more stable than the standard NF membranes.^7,17^ Another group of stable membranes that has achieved attention in the recent past and that contains sulfonic acid groups is that made of polyvinylidene fluoride grafted with sulfonated polystyrene (PVDF-*g*-PSSA). While these membranes were mainly applied as ion-exchange membranes in fuel cells as an alternative to Nafion®,^22–27^ they also have promising properties to be applied as pH-resistant NF membranes. Indeed, PVDF, a semi crystalline fluoropolymer, provides the membrane with a stable backbone as a consequence of its excellent thermal and chemical stability.^28^ However, PVDF membranes as such cannot be used to retain salts as they cannot be prepared with sufficiently high retentions for small molecules *via* conventional phase inversion.^29–32^ Moreover, PVDF is unstable at high pH (pH > 12) as bases can dehydrofluorinate the polymer.^28,33^ On the other hand, Nafion® (a sulfonated fluoropolymer), while stable in a broad pH range, is not suitable for NF as it is too dense to allow water to pass through the membrane under a pressure gradient at acceptable rates. Moreover, membranes with asymmetric structure cannot be prepared from Nafion®.

In the present work, a strategy is developed to extend the use of PVDF to the NF range: the rather large pores of PVDF membranes obtained through the process of phase inversion, were grafted with polystyrene to create a denser structure inside the original pores. The subsequent introduction of sulfonic acid groups into this polystyrene (PSSA) has the purpose of further increasing the retention of salts through Donnan exclusion. In addition to good thermal stability, the obtained PVDF-*g*-PSSA membranes are expected to display a wide pH-stability because the backbone is already dehydrofluorinated and the presence of the grafted polystyrene can prevent further attacks of a base on the PVDF backbone. The performance of these PVDF-*g*-PSSA membranes was investigated by means of filtration experiments using aqueous solutions of dyes of different size, as well as mono- and divalent salt solutions. The pH-stability of the membranes was evaluated by repeating the same filtration experiments after long-term exposure to extreme pH-conditions (pH = 0–14). The main novelty of this work lies in the new application of the PVDF-*g*-PSSA, which was so far limited to air-drying and fuel cells, and is extended here to pH-resistant nanofiltration. Additionally, the synthesis method commonly applied in literature to obtain these membranes was modified and improved for our specific application. The grafting time was reduced from 24 to 6 h and additional grafting steps were performed to increase the salt retention.

## 2. Materials and methods

### 2.1 Materials

PVDF powder (MW = 534 000 g mol^−1^), styrene, 1,2-dichlorethane, sulfuric acid (95–98%), benzoyl peroxide (BPO) and chlorosulfonic acid (70%) were purchased from Sigma-Aldrich and used without further modification. Bengal Rose (MW = 973.67 g mol^−1^) and Rhodamine B (MW = 479.02 g mol^−1^) were purchased from Fluka. Magnesium sulfate (MgSO4, ≥97%), sodium chloride (NaCl) and potassium hydroxide (KOH) were purchased from Sigma Aldrich. *N*-methyl-2-pyrrolidone (NMP), tetrahydrofuran (THF), chloroform, 1,2-dichloroethane and ethanol were obtained from Acros (analytical grade).

### 2.2 Membrane preparation

#### 2.2.1 PVDF membranes

**Scheme 1:**
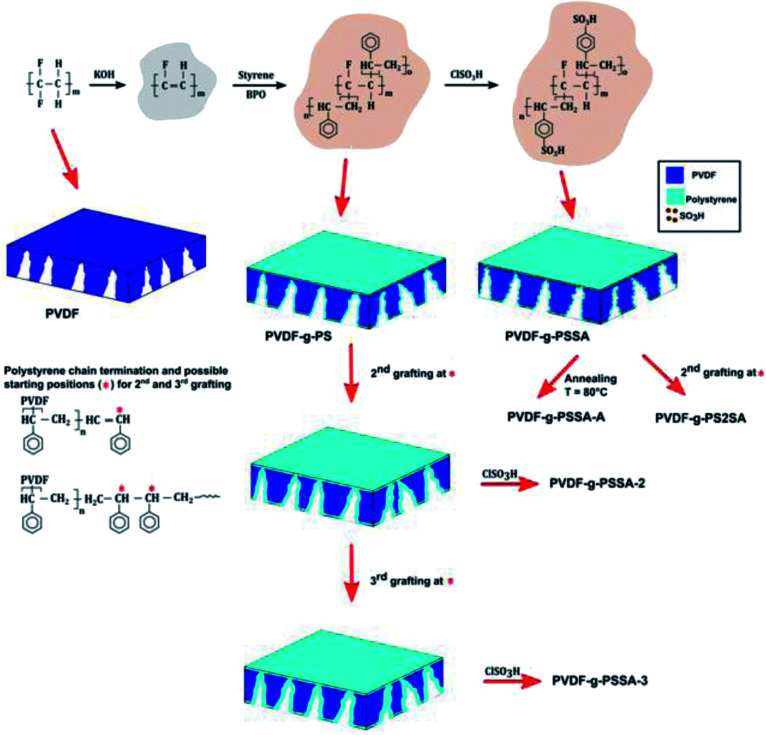
Schematic representation of the synthesis method of the different PVDF-*g*-PSSA membranes prepared and tested in this work.

The pristine PVDF membranes were prepared by phase inversion from PVDF/NMP/THF casting solutions with 15, 18 or 20 wt% PVDF concentrations. The NMP/THF ratio was kept constant at 80/20. The solution was cast onto a glass plate (40 × 60 cm) at a 250 μm wet thickness by using a lab-made casting knife (width 8 cm). After an evaporation period of 15 s, the membrane was immersed into distilled water for about 15 min to allow phase-inversion.^32^ The membranes were then washed three times with ethanol (each washing step took 2 h) to remove all water. Afterwards, they were immersed into a 1 M KOH solution in ethanol for 60 min at room temperature, which generates double bonds in the membrane matrix by eliminating H and F atoms from the PVDF backbone. These double bonds were the starting point for the grafting step. After thoroughly washing the membranes with distilled water until neutral pH, they were immersed in the grafting solution, which was composed of 80 vol% styrene, 20 vol% THF and the radical initiator BPO (3.75 × 10^−3^ g mL^−1^). The grafting reaction was performed at 60 °C for 6 h. The membrane was then extracted in a reflux setup with chloroform overnight at 120 °C to remove unreacted monomer and uncoupled polystyrene. The resulting membrane is called PVDF-*g*-PS, or PVDF grafted with polystyrene. Finally, the polystyrene graft was sulfonated completely through immersion in a 1 M solution of chlorosulfonic acid in 1,2-dichloroethane for 24 h to obtain a membrane with a negatively charged backbone. The sulfonated membranes were thoroughly rinsed with THF and distilled water, and then stored in distilled water (Scheme 1). The final membrane will be further referred to as PVDF-*g*-PSSA, where SA stands for the sulfonic acid groups, which are incorporated during the sulfonation step on polystyrene. As reference material, a membrane was also prepared in the absence of styrene during the grafting to verify whether the change in performance is indeed a consequence of the PS grafting and not of the high temperature treatment in the presence of THF or chloroform. This membrane is called PVDF-*g*-SA.

#### 2.2.2 Post-synthesis modifications

To further enhance the salt retention, two post-synthesis modifications were performed:

##### Annealing

The membrane was heated at 80 °C in a water bath for 15 min^32,34^ (referred to as PVDF-*g*-PSSA-A, Scheme 1).

##### Extra grafting

The membrane was submitted to one (referred to as PVDF-*g*-PSSA-2) or two (referred to as PVDF-*g*-PSSA-3) additional grafting steps (Scheme 1), either before (PVDF-*g*-PSSA-2 and PVDF-*g*-PSSA-3) or after the sulfonation (PVDF-*g*-PS2SA) of the membrane.

### 2.3 Filtration experiments

The membrane performance was tested in a high-throughput filtration module with a water feed and a dye (Rose Bengal or Rhodamine B, both 35 μM) and/or a salt (NaCl or MgSO_4_, both 1 g L^−1^). Rose Bengal (RB) and NaCl were permeated simultaneously. The active membrane area was 2.01 × 10^−4^ m^2^. The setup allowed simultaneous dead-end filtrations of eight membranes under the same operating conditions. In order to minimize concentration polarization, the feed solution was continuously stirred at 400 rpm. An N_2_ pressure of 10 bar was supplied for the pristine PVDF membranes and 20–30 bar for the modified PVDF membranes. To obtain reproducible results, at least three coupons of each membrane were tested. The presented results are the average values; the standard deviation is included in the tables and figures. Measurements of permeance and retention were performed after equilibrium was established.

The membrane permeance (*L*_p_) was calculated using:
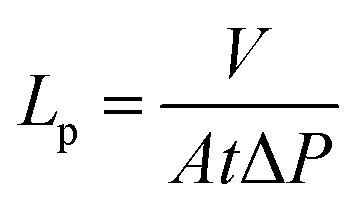
where *V* is the permeate volume (L), *A* is the membrane area (m^2^), *t* is the time (h) and Δ*P* is the applied pressure (bar).

The retention was calculated using:*R* = ((*c*_f_ − *c*_p_)/*c*_f_) × 100%where *c*_f_ and *c*_p_ are the feed and the permeate concentration, respectively. Dye concentrations were measured on a UV-1800 UV-vis spectrophotometer (Shimadzu). Salt concentrations were measured with a Consort multi parameter analyzer C3010 conductivity meter.

### 2.4 Scanning electron microscopy (SEM) and energy-dispersive X-ray (EDX) spectroscopy

The membrane cross-sections and surfaces were observed with scanning electron microscopy (SEM) using a Philips XL30 FEG SEM, *i.e.* a semi-in-lens type microscope with a cold field emission electron source. The cross-section were obtained after breaking the membranes under liquid nitrogen. The samples, top and cross sections, were glued on a sample holder, dried and coated with a thin carbon layer *via* a high resolution sputter coater (JEOL JFC-1300). Energy-dispersive X-ray (EDX) spectroscopy was used in combination with SEM to visualize the distribution of the sulfonic groups throughout the membrane. The elemental distribution was recorded with an Edax EDX detector with an ultra-thin window from Phoenix. Data analysis was performed with Genesis spectrum version 6.46. EDX maps were made based on a total of 30 scans with the SEM operated at 10 kV, at 650× magnification and with spot size 3.

### 2.5 Attenuated total reflectance Fourier transform infrared spectroscopy (ATR-FTIR)

The chemical structure of the sample was analyzed by Attenuated Total Reflectance Fourier Transform Infrared (ATR-FTIR) spectroscopy using a VARIAN 620-IR FT-IR spectrometer (Agilent). Prior to measurements, the membranes were dried for 45 min in an oven at 100 °C to remove excess water.

### 2.6 Contact angle measurements

Contact angle measurements were conducted to investigate the hydrophilic/hydrophobic character at different stages during the membrane synthesis. The measurements were conducted with a high resolution camera linked to a computer (Drop Shape Analysis system, DSA 10 Mk2 from Kruss). Prior to the measurements, the membrane samples were dried overnight at 100 °C.

### 2.7 Zeta-potential measurements

Membrane charge properties were characterised from streaming current measurements performed with a SurPASS electrokinetic analyzer (Anton Paar GmbH, Austria) equipped with an adjustable-gap cell. All measurements were carried out by setting the distance between the two membrane coupons in the measuring cell to 100 ± 5 μm. Streaming current was measured with a pair of Ag/AgCl electrodes by applying pressure ramps of 300 mbar. Visiolab software was used for data analysis (zeta potential determination). All experiments were performed with 10^−3^ M KCl background solutions at room temperature (25 ± 2 °C) following the experimental protocol described by Mouhoumed *et al.*^35^ Membranes were first equilibrated with the background solution at pH ≈ 9 (pH was adjusted with 0.1 M KOH) and then streaming current measurements were performed by progressively decreasing the pH down to about 3 by additions of 0.1 M HCl.

### 2.8 Atomic force microscopy (AFM)

Membrane topographic images were acquired under water (TM Direct Drive holder) using a Dimension 3100 device (Bruker) in tapping mode with standard NC cantilevers (Nanosensors, PPP-NCHR, resonant frequency in air ∼320 kHz). The reported roughness values are the average of at least three different locations on each membrane sample. All samples were measured over an area of ≈25 μm^2^.

### 2.9 Tensile strength tests

The mechanical properties of the dried membranes were investigated with an INSTRON 5943 setup applying a cross-head elongation speed of 0.1 to 1 mm min^−1^. The membranes were cut in samples of 10 by 20 mm. All samples were investigated under ambient conditions.

### 2.10 X-ray photoelectron spectroscopy (XPS) measurements

Spectra were recorded on a Kratos Axis Supra X-ray Photoelectron Spectrometer employing a monochromated Al Kα (*hν* = 1486.6 eV, 8 mA emission) X-ray source, hybrid (magnetic/electrostatic) optics with a slot aperture, hemispherical analyser, multichannel plate and delay line detector (DLD) with a take-off angle of 90°. The analyser was operated in fixed analyser transmission (FAT) mode with survey scans taken with a pass energy of 160 eV and high resolution scans with a pass energy of 20 eV. All scans were acquired under charge neutralization conditions using a low energy electron gun within the field of the magnetic lens. The resulting spectra were processed using CasaXPS software. Binding energy was referenced to aliphatic carbon at 285.0 eV. High resolution spectra were fitted using the “LF(*α*, *β*, *w*, *m*)” lineshape corresponding to a numerical convolution of Lorentzian functions (with exponents *α* and *β* for the high binding energy and low binding energy sides) with a Gaussian (width *m*) and inclusion of tail-damping (*w*) to provide finite integration limits. Details of this lineshape function are available in the CasaXPS documentation online.^36^ Empirically determined relative sensitivity factors provided by Kratos Analytical Ltd (Manchester, UK) were used for quantification. Use of these relative sensitivity factors does not account for any attenuation due to overlayers or other surface contamination and assumes a uniform depth distribution of elements within the information depth of the sample. Matrix effects are also discounted.^37,38^ Reported errors in atomic compositions are based on Monte Carlo simulations of the stability of fitting the XPS data with respect to noise and represent the minimum error in the values. Doublets due to non-zero orbital angular momentum were modelled using a fixed ratio of component peaks corresponding with the degeneracy of the total angular momentum states for that set of orbitals.

It should be noted that various elements were observed in the XPS analysis which were not expected to be present in the membranes (*e.g.* N, Na, Si, Cl, Ca). Since N, Si, Cl and Ca were not detected by EDX (which has a deeper analysis than XPS, due to the more energetic electron beam), their identification by XPS is ascribed to surface contamination of the material. As a consequence, the XPS data were employed only for the qualitative analysis in Section 3.2 and not for quantitative analysis.

### 2.11 Long-term pH-stability tests

After an initial filtration experiment, the used membrane coupons were immersed in 100 mL of either 1 M HCl or 1 M NaOH aqueous solution at room temperature for one week. Subsequently, the membranes were rinsed with distilled water until a neutral pH was obtained. Hereafter the membrane performance was evaluated again using the very same membrane coupons to gain a first indication of their pH-stability. The weight change after one week was also recorded as an extra indicator of the pH-stability.

## 3. Results and discussion

A set of different PVDF-*g*-PSSA membranes was synthesized according to Scheme 1. The grafting is anticipated to narrow the pores size of the pristine PVDF-membrane, generating a denser membrane with a better retention for smaller molecules. Creation of a very thin PSSA-layer also on top of the original membrane surface is expected to happen simultaneously. The negative charges on the grafted polymer chains, created by sulfonation, are expected to result in higher salt retentions as a consequence of Donnan exclusion.^1^ In an attempt to still enhance the salt retention, two different post-synthesis methods were investigated to further densify the membranes. The membrane was either annealed after the sulfonation (PVDF-*g*-PSSA-A), or grafted a second (PVDF-*g*-PSSA-2) and a third (PVDF-*g*-PSSA-3) time prior to performing the sulfonation. As an extra test, a second grafting step was also performed after the sulfonation step (PVDF-*g*-PS2SA). This membrane was not sulfonated a second time.

### 3.1 Pristine PVDF membrane

**Fig. 1:**
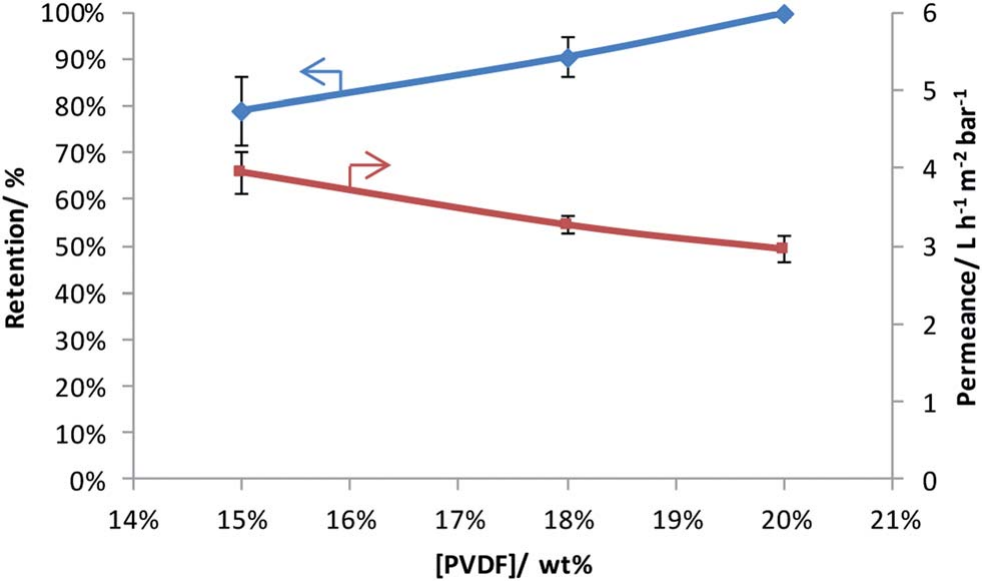
Rose Bengal retention and water permeance as a function of weight percentage of PVDF in the casting solution.

Three PVDF-membranes were prepared by phase inversion from solutions with different PVDF concentrations and their performance was investigated by filtering an aqueous RB solution (Fig. 1). As anticipated, the results show that the retention increases and the permeance decreases with the PVDF concentration in the casting solution.^32,39,40^ Since the best retention of RB was achieved with 20 wt% PVDF in the casting solution, only this membrane was further modified through grafting and sulfonation to render it more selective.

**Fig. 2:**
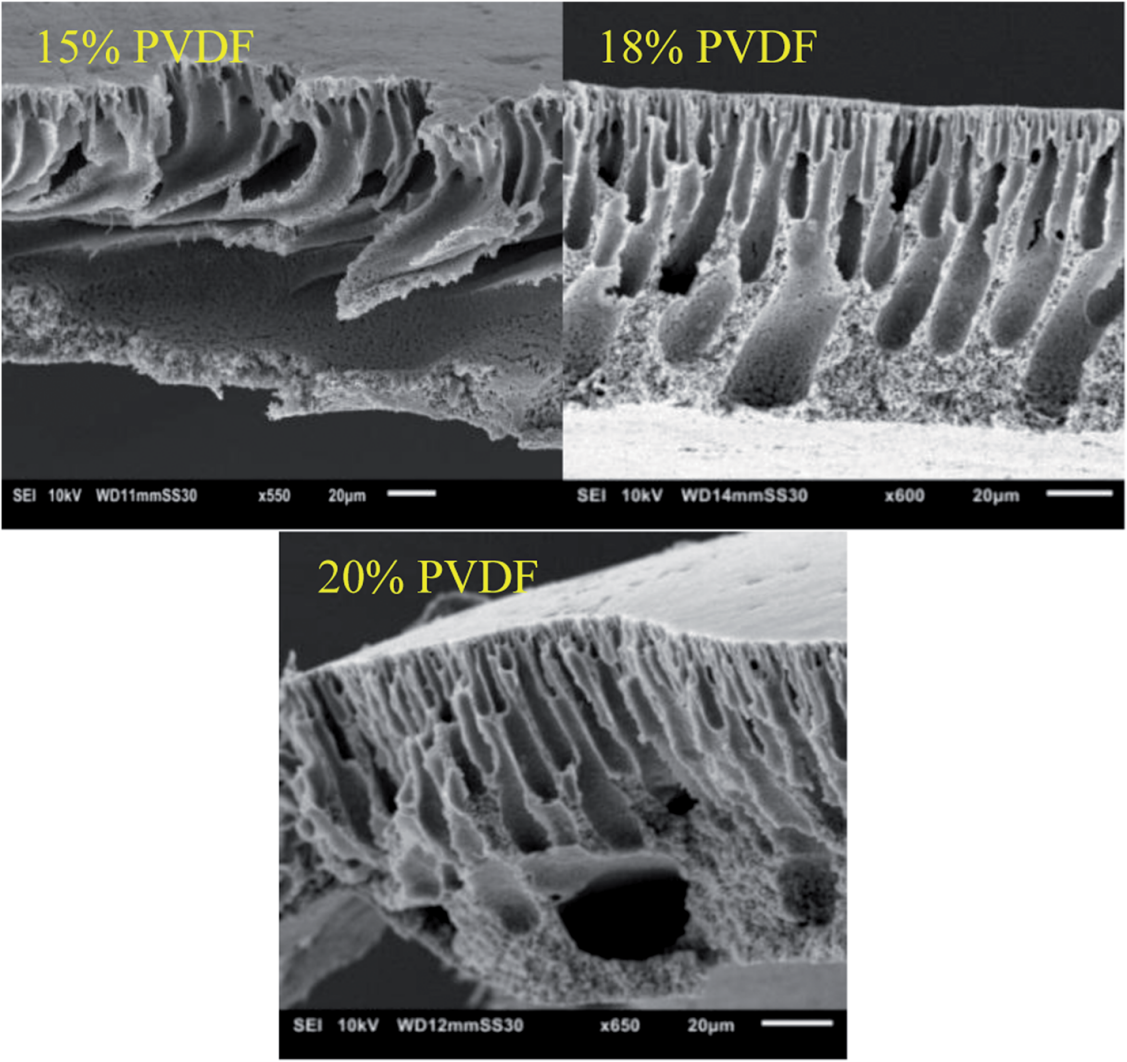
SEM images of the cross-sections of the membranes prepared with different weight percentages of PVDF in the casting solutions.

Based on the SEM images (Fig. 2), no clear effect of the casting film concentration on the membrane structure and morphology could be observed as all membranes clearly contain a dense top layer of similar thickness and a sublayer with fingerlike macrovoids. However, based on the increased performance at higher concentration of PVDF in the casting solution, smaller pores in the selective layer of these membranes can be expected.

### 3.2 PVDF-*g*-PSSA membranes

**Fig. 3:**
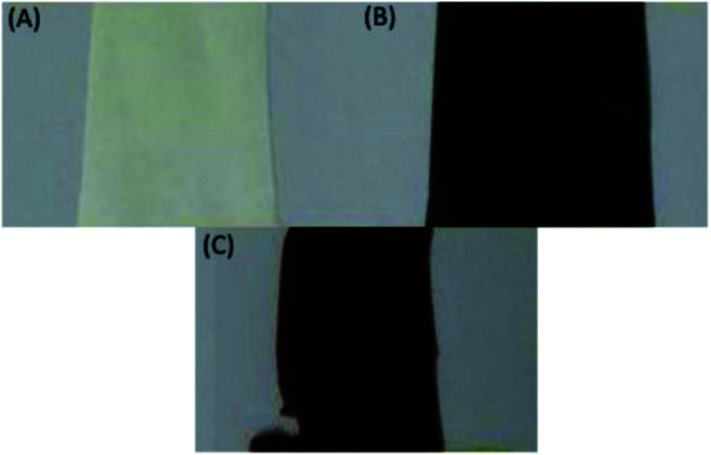
Photographs of the membrane (A) parent, (B) after dehydrofluorination and (C) after grafting and sulfonation.

The selected PVDF membrane was dehydrofluorinated through treatment with a KOH solution. The success of the dehydrofluorination step was monitored qualitatively by mere visual observation: after dehydrofluorination, the membrane turned black (Fig. 3). This follows from the formation of a more conjugated system as a consequence of the formation of double and even triple bonds after HF elimination. The black color indicates that the conjugated system is long enough to cause such darkening and/or that a substantial amount of triple bonds is present. After the grafting, the color of the membranes changed to brown, which remained the same also after the sulfonation step (Scheme 1, Fig. 3). This indicates the disappearance of the triple bonds and of the majority of the double bonds.

To investigate the influence of the grafting and sulfonation on the hydrophilicity of the membranes, contact angle measurements were performed. For the pristine PVDF membrane, a contact angle of 75° was recorded, which increased to 85° after grafting the membrane with polystyrene (PVDF-*g*-PS) and decreased to almost 0° for the sulfonated membranes (PVDF-*g*-PSSA). The increase in contact angle after grafting proves the more hydrophobic nature of polystyrene compared to PVDF. Furthermore, the sulfonation is considered successful since the contact angle decreases to nearly 0°, indicating a very hydrophilic character of the membrane, which is caused by the presence of the hydrophilic sulfonic acid groups. The value of close to zero is given for the PVDF-*g*-PSSA membranes, while it was actually not possible to measure the real value with the camera as the droplet immediately spread out over the surface once it came into contact with it, suggesting a very hydrophilic membrane.

**Fig. 4:**
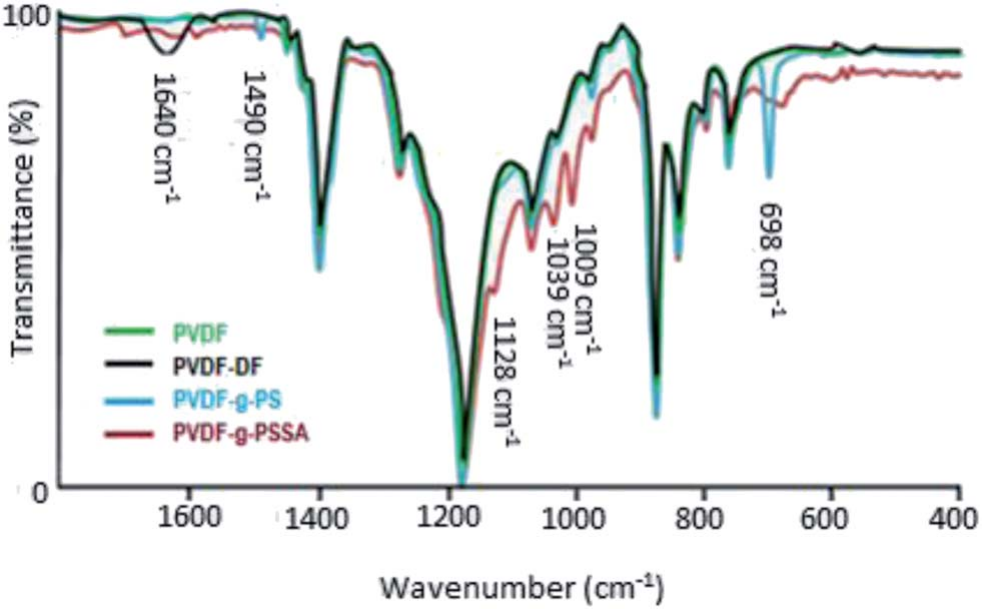
ATR-FTIR spectra of PVDF, PVDF-DF (after dehydrofluorination), PVDF-*g*-PS and PVDF-*g*-PSSA.

ATR-FTIR spectra were recorded at different stages in the synthesis to confirm the visual observations and to further verify whether PVDF was successfully grafted with polystyrene and subsequently sulfonated (Fig. 4). Besides the characteristic peaks of pristine PVDF [C–H bending (1400 cm^−1^), C–F stretching (1180 cm^−1^), C–H wagging (870 cm^−1^) and C–F bending (830 cm^−1^)],^41^ one additional, broad peak appears after the dehydrofluorination step at 1640 cm^−1^, which can be assigned to the C=C bond stretching modes. After the grafting, two additional peaks appear at 698 and 1490 cm^−1^, which are assigned to the out of plane ring deformation of mono-substituted phenyl groups^42^ and the C=C in-plane stretching vibration modes of the phenyl rings,^43^ respectively. The bond at 1640 cm^−1^ disappears upon grafting, confirming that most of the double bonds reacted in the grafting of polystyrene. The C≡C bond (2100–2260 cm^−1^) could not be observed in the IR spectra because of its weak dipole and the weak IR signal as a result. As a consequence, IR is not a suitable technique to detect alkyne bonds, especially when they are symmetrical (even weaker signal).^34^ After sulfonation, the peaks at 698 and 1490 cm^−1^ diminish and/or shift since the phenyl rings are no longer mono-substituted. Furthermore, three additional peaks appear, which can be assigned to the symmetric (1009 and 1039 cm^−1^) and the asymmetric (1128 cm^−1^) stretching vibration of the S=O bond.^44^ A complete sulfonation (100%) is expected to have taken place after 24 h using a 1 M solution of chlorosulfonic acid. This was concluded based on our previous work where the focus was on improving the ion exchange capacity and thus the conductivity of these membranes. Since it was observed that longer sulfonation or harsher conditions did not result in increased ion exchange capacities, it was concluded that the sulfonation reaction was complete.

**Fig. 5:**
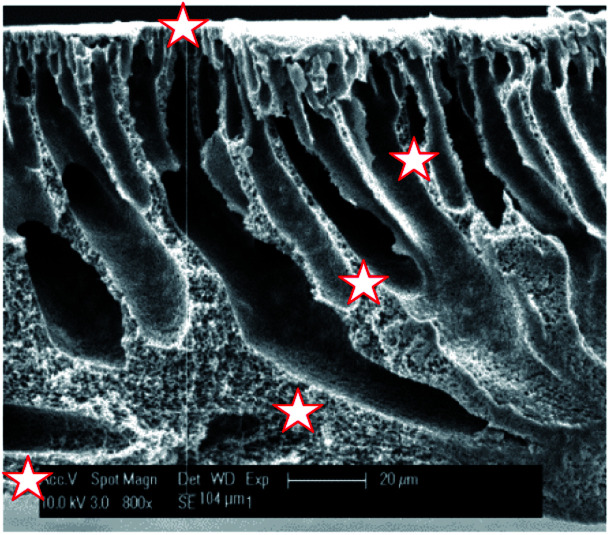
SEM image of the cross-section of PVDF-PSSA-1 membrane with the positions where EDX was measured being marked with a star.

SEM was used in combination with EDX to investigate the distribution of sulfur (and thus of the sulfonic acid groups) across the PVDF matrix (Fig. 5 and Table 1). It was found that the sulfur is distributed rather homogeneously throughout the membrane (between 3.1 and 4.2 at%), with the exception of the top zone of the membrane where only 1.6 at% of sulfur could be found. Under the applied conditions, the EDX probing is assumed to penetrate to a depth of about 1 μm. This lower sulfur content in the top layer is most likely caused by the denser nature of the top of the membrane where the narrowest pores are located, hindering the styrene grafting and the chlorosulfonic acid diffusion to the phenyl rings.

**Table 1:** Elemental composition at different distances from the skin layer (*i.e.* top layer of original PVDF membrane) as determined by EDX measurements

Distance from top [μm]	C [at%]	O [at%]	F [at%]	S [at%]
0	77	7.2	14	1.6
36	67	4.5	24	4.1
59	68	5.9	23	3.1
89	67	6.1	23	3.5
104	70	4.6	21	4.2

**Fig. 6:**
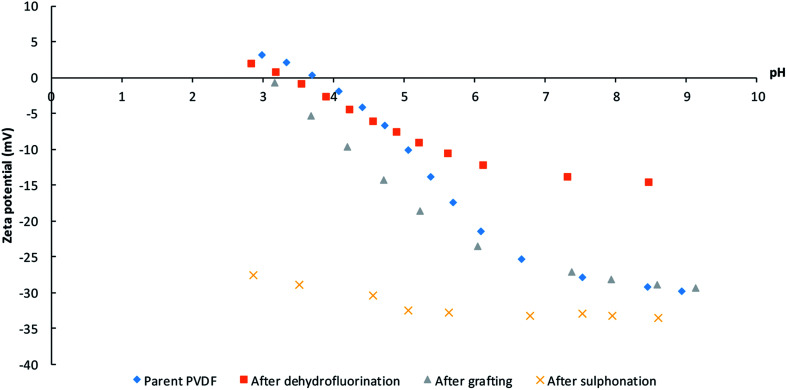
Zeta-potential measurements after the different modifications: pristine PVDF, after dehydrofluorination, after grafting and after sulfonation.

The surface charge as a function of pH after each synthesis step was investigated with zeta-potential measurements (Fig. 6). The surface of the pristine PVDF membrane surface is negatively charged until it reaches its isoelectric point at a pH of 3–4. This behavior is similar to that observed for other uncharged membranes and is attributed to the adsorption of hydroxide ions, which originate from the water self-ionisation.^45^ The KOH treatment results in a decreased tendency to coordinate hydroxide anions as a consequence of the dehydrofluorination, which introduces double (and triple) bonds along the polymer backbone and thus decreases the number of functional groups that can interact with the hydroxide anions. After grafting with polystyrene, the charge density increases to a value comparable to that of the pristine PVDF, indicating that the polystyrene modification did not introduce additional charged groups to the system.^46^ After sulfonation, the isoelectric point disappears and the negative charge density becomes almost independent of the pH of the solution, which indicates an efficient sulfonation of polystyrene as it points out to the presence of sulfonic groups that behave as strong acids.^47^ Higher and lower pH values were not measured as plateaus were already achieved and no further change in potential is expected (especially not a change in the sign of the charge).

**Fig. 7:**
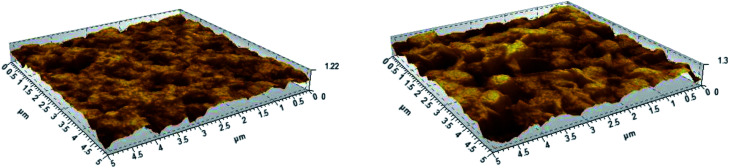
AFM images of the pristine PVDF (left) and PVDF-*g*-PSSA-3, showing the surface roughness of the membranes.

Atomic Force Microscopy (AFM) measurements were performed to determine the surface roughness of the membranes before and after grafting (Fig. 7). After grafting and sulfonation, the root mean square roughness of the membranes measured on 1 × 1 μm scans increases from 78 to 157 nm, and on 5 × 5 μm scans from 157 to 228 nm. This shows that the additional sulfonated polystyrene layer creates extra roughness, giving rise to increased surface area.

Finally, the mechanical properties of the pristine and treated (PVDF-*g*-PSSA-3) membranes were investigated with a tensile strength tester. The average tensile strength at maximum load for the pristine PVDF membrane was 3.4 MPa, in accordance with literature.^48–52^ For the PVDF-*g*-PSSA-3 membrane, an average tensile strength of 2.1 MPa was recorded, which indicates that the grafting procedure reduces the mechanical strength of the membrane. In accordance with the tensile strength, the Young's modulus increases from 5.4 MPa for the pristine membrane to 12.8 MPa for the modified membrane (PVDF-*g*-PSSA-3), which indicates that the grafted membranes are more rigid. Both the lower tensile strength and the increased rigidity upon grafting and sulfonation can be ascribed to the lower flexibility of the polystyrene chains as compared to the PVDF matrix.

The membrane performance was investigated to evaluate the effects of grafting and sulfonation (Table 2). After grafting, the membrane water permeance decreased by a factor of 15. This dramatic decrease can be related to the presence of an extra layer on top of the membrane and inside its pores, creating a denser membrane. This layer forms an extra barrier against the water permeance through the membrane. Furthermore, since polystyrene is more hydrophobic than PVDF, this layer repels water more effectively. Upon sulfonation, the hydrophilicity of the membrane increases drastically due to the presence of the polar and acidic sulfonic groups (Scheme 1). As a consequence, the water permeance increases strongly. This increase can also be partly attributed to the increased available surface area, which follows from the increased surface roughness. In addition, the salt rejection increases because of the Donnan exclusion, which stems from the introduction of ionic groups. For comparison, a membrane was also prepared in the absence of styrene during the grafting step (PVDF-*g*-SA) and its performance was investigated. The observed increase in permeance and decrease in retention of both Rose Bengal and NaCl as compared to the other membranes (Table 2) is attributed to the open structure and hydrophilic nature of PVDF-*g*-SA. This more open structure is most likely generated during the high-temperature treatment in the presence of the radical initiator, which in the absence of styrene might cause the PVDF membranes to deteriorate. Additionally, the presence of sulfonic acid groups will increase the hydrophilicity of the membranes, yielding increased permeances, which might, in the case of this more open structure, drag along more RB to the permeate side.

**Table 2:** Retention for different solutes and water permeance for membranes obtained after different steps in the synthesis method of the PVDF-*g*-PSSA membrane. Filtration conditions: operating pressure: 20–30 bar; rotation speed: 500 rpm; feed: 35 μM RB and 1 g L^−1^ NaCl in milliQ water or 35 μM Rhodamine B in milliQ water

Solute	Permeance [L h^−1^ m^−2^ bar^−1^]	Retention [%]		
		Rose Bengal	Rhodamine B	NaCl
PVDF	3.0 ± 0.5	99.9 ± 1.0	61.0 ± 0.6	17.9 ± 8.2
PVDF-*g*-PS	0.2 ± 0.1	99.9 ± 0.5	94.0 ± 0.6	18.4 ± 2.5
PVDF-*g*-PSSA^a^	4.4 ± 0.5	99.8 ± 0.3	—c	30.7 ± 0.8
PVDF-*g*-SA	5.56 ± 1.73	63.9 ± 0.4	—c	0.5 ± 0.4
PVDF-*g*-PSSA^b^	3.0 ± 0.5	99.9 ± 0.4	96.0 ± 0.8	39.7 ± 0.8

a This membrane was prepared from a 18% PVDF membrane as follows: (1) dehydrofluorination for 250 min in a 0.1 M KOH solution in ethanol; (2) grafting for 16 h at 80 °C with 80/20 vol% styrene/THF solution containing 3 × 10^−3^ g mL^−1^ BPO and (3) sulfonation for 24 h in a 0.1 M solution of chlorosulfonic acid in dichloroethane.

b This membrane was prepared according to the optimized conditions mentioned under paragraph 2.2.1.

c Not measured.

In a parallel study, the parameters of the different membrane modification steps (dehydrofluorination, grafting and sulfonation) were altered for the 20 wt% PVDF membrane and the conditions were made more extreme in an attempt to achieve NaCl retentions above 30% and thus to bring the membrane performance to the level of tighter NF. Based on visual observations, the grafting step was reduced from 18 to 6 h since a complete solidification (*i.e.* the styrene present in the reaction solution became completely polymerized after 6 h, and as a consequence the membrane itself was stuck inside a polystyrene block) of the reaction environment was already observed after 6 h. Aiming at a higher degree of grafting and sulfonation and thus increased salt retentions, the concentrations of KOH, BPO and chlorosulfonic acid were increased from 0.1 to 1 M, from 3 to 3.75 g mL^−1^ and from 0.5 to 1 M, respectively. Although a certain improvement was observed (last entry in Table 2), the salt retention remained rather low even after applying these more extreme conditions.^6,53^ Since both Rose Bengal and the salts are ionic compounds, it is possible that an increased retention is merely a consequence of the addition of the ionic groups through the sulfonation step and of the related Donnan exclusion principle. To verify this and to investigate whether the presence of the higher resistance due to the additional graft layer on top of the membrane and in the pores also plays a role, the influence of the membrane modification on the retention of a compound containing a large cation (Rhodamine B), which should permeate faster in the presence of negatively charged sulfonic groups, was also investigated after each step in the synthesis. As shown in Table 2, the retention of Rhodamine B increases up to around 95% after the grafting and sulfonation. It can also be observed that a high Rhodamine B retention was already achieved prior to the sulfonation step (*i.e.* for PVDF-*g*-PS) clearly indicating that the retention is also strongly enhanced by the presence of the additional graft layer.

**Fig. 8:**
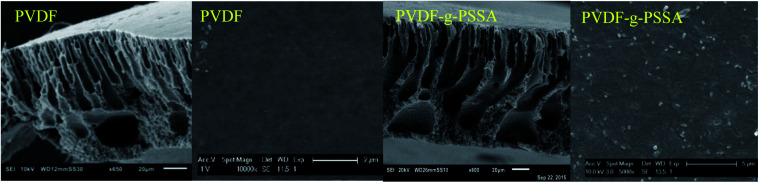
SEM images of the cross-sections and surfaces of the pristine PVDF membranes and of the PVDF-*g*-PSSA membranes.

The influence of the membrane modifications on the membrane morphology can be observed in Fig. 8. While no clear changes in the morphology can be seen from the SEM pictures of the membrane cross-sections, the images of the surface of PVDF-*g*-PSSA shows some new features (appearing as lighter domains) compared to the pristine PVDF, which suggest that the PVDF surface was successfully covered with grafted sulfonated polystyrene. On the contrary to what the lighter domains suggest, this layer is homogeneously formed across the membrane (although possibly with variable thickness), as evidenced by Fig. S1 & S2.†

**Fig. 9:**
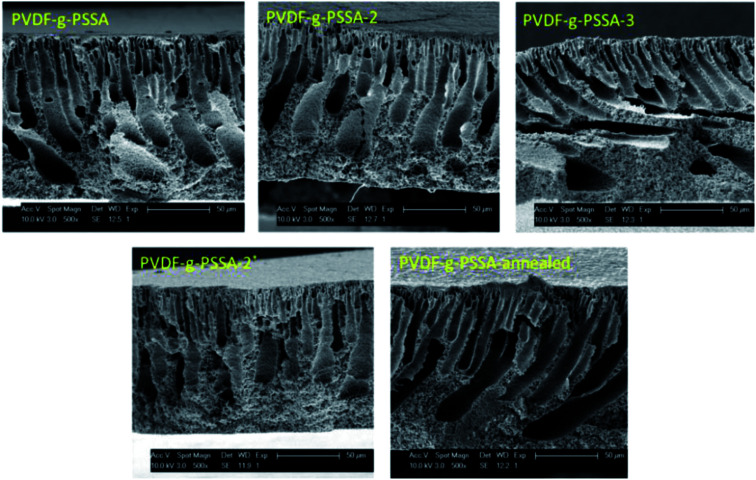
SEM images of the cross-sections of the different PVDF-*g*-PSSA membranes.

**Fig. 10:**
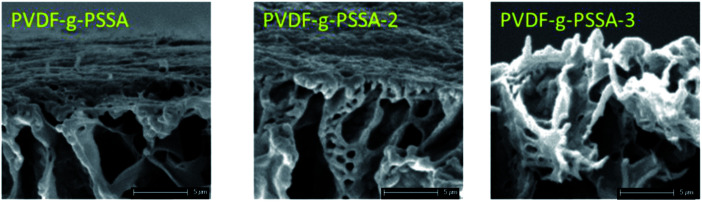
Close-up SEM images of the dense layer of the PVDF-*g*-PSSA membranes with different number of grafting steps.

**Fig. 11:**
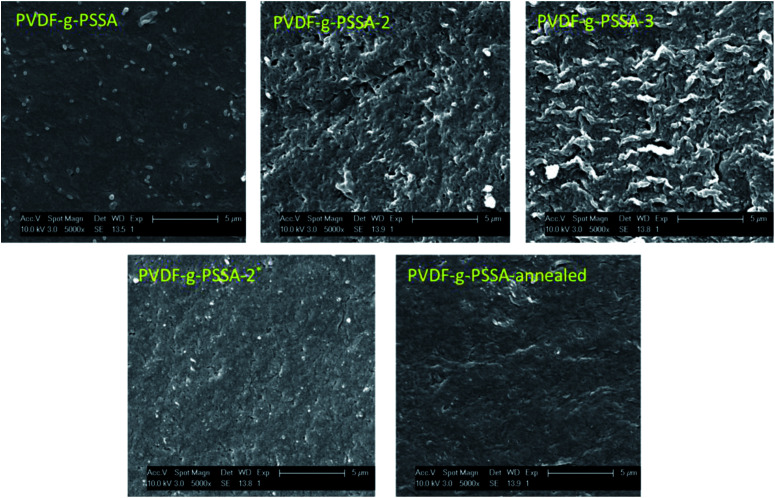
SEM images of the surface areas of the different PVDF-*g*-PSSA membranes.

Using these harsher reaction conditions and starting from the 20 wt% PVDF, the polystyrene grafting process was repeated a second and third time, prior to performing the sulfonation step, to further improve the salt rejection. This would occur through an attack by BPO at the sites marked with an * in Scheme 1, generating free radical sites that can be further grafted with polystyrene. This would result in the creation of extended and/or branched polystyrene chains, which would lead to thicker and/or denser (possibly cross-linked) grafted layers on the PVDF top layer and/or inside the pores. After a second and a third grafting step and subsequent sulfonation, the salt retention further increased while the permeance decreased (Table 3), due to the formation of a denser and thicker layer on top of the membrane and inside the membrane pores. Indeed, it can be expected that the second and third grafting step will take place wherever the initiator and the monomers can still reach, hence filling up the remaining voids with extra polystyrene or extending the polystyrene chains that lie on top of the dense layer. However, this hypothesis could not be confirmed by the SEM images, which all look quite similar (Fig. 9). A close-up of the dense layer of the PVDF-*g*-PSSA membranes with different number of grafting steps could also not give closure on the expected formation of a thicker polystyrene top layer (Fig. 10). On the other hand, when the surface layers of the membranes were analyzed, the increase in the fraction of polystyrene could tentatively be inferred from the increase in the number of surface features (white areas in Fig. 11), suggesting that the degree of grafting was increased by performing several separate grafting steps of 6 h. Furthermore, as the membranes became more and more brittle upon drying with the increasing number of grafting steps, some cracks appeared for PVDF-*g*-PSSA-2 and -3. More evidence for the increase in grafting degree can be found in Fig. S3,† where an increase in the number of grafting steps results in an increased intensity of the bands assigned to the phenyl rings (698 and 1490 cm^−1^).

**Table 3:** Retention and permeance of PVDF-*g*-PSSA membranes after further modifications. Filtration conditions: operating pressure: 20–30 bar, rotation speed: 500 rpm and feed: 35 μM RB and 1 g L^−1^ NaCl in milliQ water or 1 g L^−1^ MgSO4 in milliQ water

	Permeance [L h^−1^ m^−2^ bar^−1^]	Retention [%]		
		Rose Bengal	NaCl	MgSO_4_
PVDF-*g*-PSSA	3.0 ± 0.5	99.9 ± 0.4	39.7 ± 0.8	61.3 ± 0.7
PVDF-*g*-PSSA-2	2.4 ± 0.3	97.0 ± 4.0	48.3 ± 2.6	66.4 ± 0.8
PVDF-*g*-PS2SA	2.1 ± 0.2	61.2 ± 6.2	08.8 ± 1.2	19.6 ± 1.1
PVDF-*g*-PSSA-3	2.4 ± 0.6	99.4 ± 1.3	57.1 ± 0.4	77.4 ± 0.5
PVDF-*g*-PSSA-A	2.6 ± 0.8	81.8 ± 7.4	06.0 ± 2.2	14.8 ± 1.5

If the second grafting step is performed after the sulfonation step and without further sulfonation afterwards, a decrease in retention was observed, both for Rose Bengal and NaCl (Table 3, 3^rd^ entry). This can be explained based on the high hydrophobicity of polystyrene and thus of the outer layers of the membrane. While handling this membrane, we noted that it was rather brittle. So it can be assumed that several microcracks were created when increasing the trans-membrane pressure during the filtration experiments. Finally, a decrease in performance was observed also for the annealed membranes, which contradicts the knowledge available in the literature. Indeed, it would be expected that the salt rejection increased after annealing since this treatment causes void shrinkage through a better chain stacking.^32^ Since each membrane was tested 3 times and the performance of the annealed membrane was compared with the pristine PVDF-*g*-PSSA membrane, an experimental error cannot be at the origin of this unexpected behavior. A possible explanation is that the sulfonic groups cluster during the annealing step, generating some kind of ion channels. This phenomenon is well known for the related Nafion® type membranes.^54^ These channels would facilitate transport of charged components through the membrane with a lower retention as result.

**Fig. 12:**
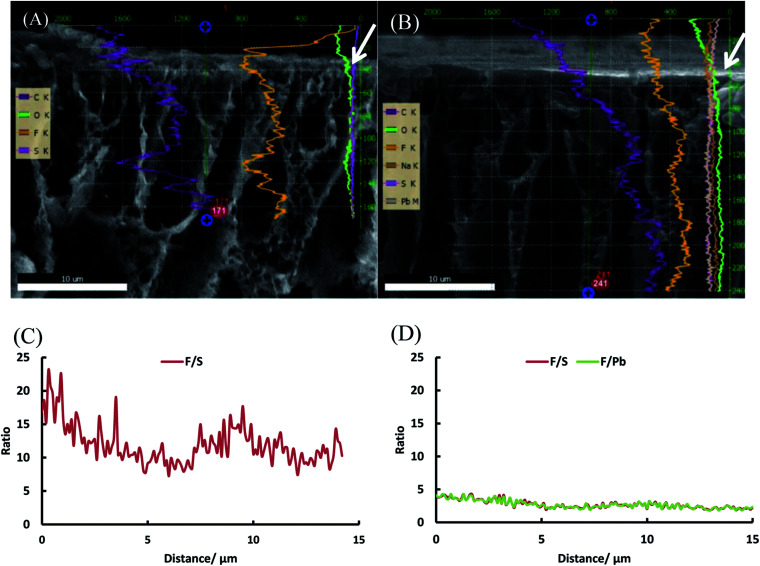
SEM images of the top layers of PVDF-*g*-PSSA-1 (A) and PVDF-*g*-PSSA-3 (B) with the EDX line scans superimposed and F/S and F/Pb ratio as a function of the distance from the top for PVDF-*g*-PSSA-1 (C) and PVDF-*g*-PSSA-3 (D). The white arrows in figure A and B indicate the point of origin (0 μm) from where the F/S and F/Pb ratios are determined in figure C and D.

To further investigate our hypothesis that extra grafting steps result in an increased thickness of the polystyrene layer on top of the dense layer, a line scan was performed with EDX on the SEM image of the top layer (first 30 μm) of PVDF-*g*-PSSA-1 and PVDF-*g*-PSSA-3 (see Fig. 12). As the polystyrene layer does not contain fluorine atoms, the fluorine to sulfur (F/S) ratio should be lower in this layer. So for PVDF-*g*-PSSA-3, a thicker layer with a lower F/S ratio is expected than for PVDF-g-PSSA-1. For PVDF-*g*-PSSA-3 an attempt was made to increase the contrast between the pristine PVDF membranes and the grafted layers by staining the membrane with PbNO_3_, as it is expected that the Pb^2+^ ions will only coordinate with the sulfonic groups,^55^ which are available in the polystyrene layer. This is confirmed by the trends in Fig. 12D, where the F/S and F/Pb ratios follow a very similar trend. For PVDF-*g*-PSSA-3, also the F/Pb ratio can thus be used to differentiate between the PVDF top layer and the grafted layer. As the staining was not performed with MilliQ water, sodium could also be detected in the EDX spectrum of this sample as these ions can also coordinate with the sulfonic groups. Unfortunately, the grafted layers on top of the membrane cannot be clearly distinguished in Fig. 12A and B and also not by using the F/S and/or F/Pb ratios (Fig. 12C and D). This is most likely related to the limited spatial resolution of the EDX detector, which is around 0.1 μm. TEM was used to further analyze the thickness of the grafted layer, but also this attempt was unsuccessful as the membranes were not stable under the intense electron beam that was required in the TEM instrument. Altogether, no proof could be found for this hypothesis with the available characterization techniques. This might mean one of the following: (1) either the growth of the polystyrene chains only occurs inside the pores and is thus impossible to visualize with the applied techniques or (2) other phenomena, also giving rise to enhanced salt retentions, are occurring simultaneously with the extension of the polystyrene layer. These phenomena may include: formation of additional polystyrene chains, cross-linking and annealing, all of which could result in a densification of the membrane. All of these steps can possibly take place during the further grafting and cleaning steps with chloroform. First of all, even if according to the IR spectra of PVDF-*g*-PSSA-1 all double bonds (which were originally created in PVDF-DF), have reacted during the first grafting step, it is possible that some minor fraction of double bonds (below the IR-detection limit) could still be available for the formation of extra polystyrene chains during the second and third grafting step, giving rise to a denser structure. Furthermore, as practically no double bonds remain after the first grafting, further reaction with styrene could possibly start from the polystyrene branches themselves (see Scheme 1, red stars). It is therefore possible that the new growing polystyrene chains connect the already existing polystyrene chains, forming cross-links, thus densifying this layer. Finally, it is also possible that further grafting and cleaning steps result in the removal of residual styrene oligomers, thus inducing rearrangement of the polystyrene layer into a denser structure. The former two phenomena would explain the increased chemical resistance (lower solubility in DMSO) of PVDF-*g*-PSSA-2 and -3 *vs.* PVDF-*g*-PSSA-1. All of these phenomena together would explain the observed changes in filtration performance (increased retention *vs.* decreased permeance).

To further investigate the distribution of the grafted layer throughout the membrane, an EDX mapping was performed on the cross-section and the top layer of the PVDF-*g*-PSSA-3 membrane (see Fig. S1 & S2†). From these images it can be observed that sulfur is homogeneously distributed throughout the membrane, which is a proof of homogeneous grafting and sulfonation throughout the whole membrane. Finally, XPS was used to investigate the surface composition of the different membranes (Table 4 and S1 and Fig. S4 to S7†). The most important conclusions that can be drawn from XPS are: (1) the relative fluorine fraction decreases after grafting and sulfonation, which is caused both by the dehydrofluorination and by the presence of the fluorine-free groups introduced by the grafting of the polystyrene; (2) the sulfur content is higher in the PVDF-*g*-PSSA membranes compared to the pristine membrane, indicating a successful sulfonation and (3) the S 2p signal is assigned to S(vi) species (Fig. S3 to S6†), which confirms the incorporation of sulfonic acid groups.^56,57^

**Table 4:** Surface composition of different membranes as determined by XPS. Values for S and O for PVDF are due to sample contamination

Sample	at% C	at% F	at% S	at% O
PVDF	64.20 ± 0.23	21.46 ± 0.13	0.41 ± 0.04	9.90 ± 0.15
PVDF-*g*-PSSA-1	62.56 ± 0.19	14.47 ± 0.12	1.09 ± 0.04	16.34 ± 0.12
PVDF-*g*-PSSA-2	65.83 ± 0.18	14.87 ± 0.10	2.56 ± 0.04	13.89 ± 0.13
PVDF-*g*-PSSA-3	64.18 ± 0.20	18.25 ± 0.10	1.87 ± 0.04	12.96 ± 0.12

### 3.3 pH-stability

**Fig. 13:**
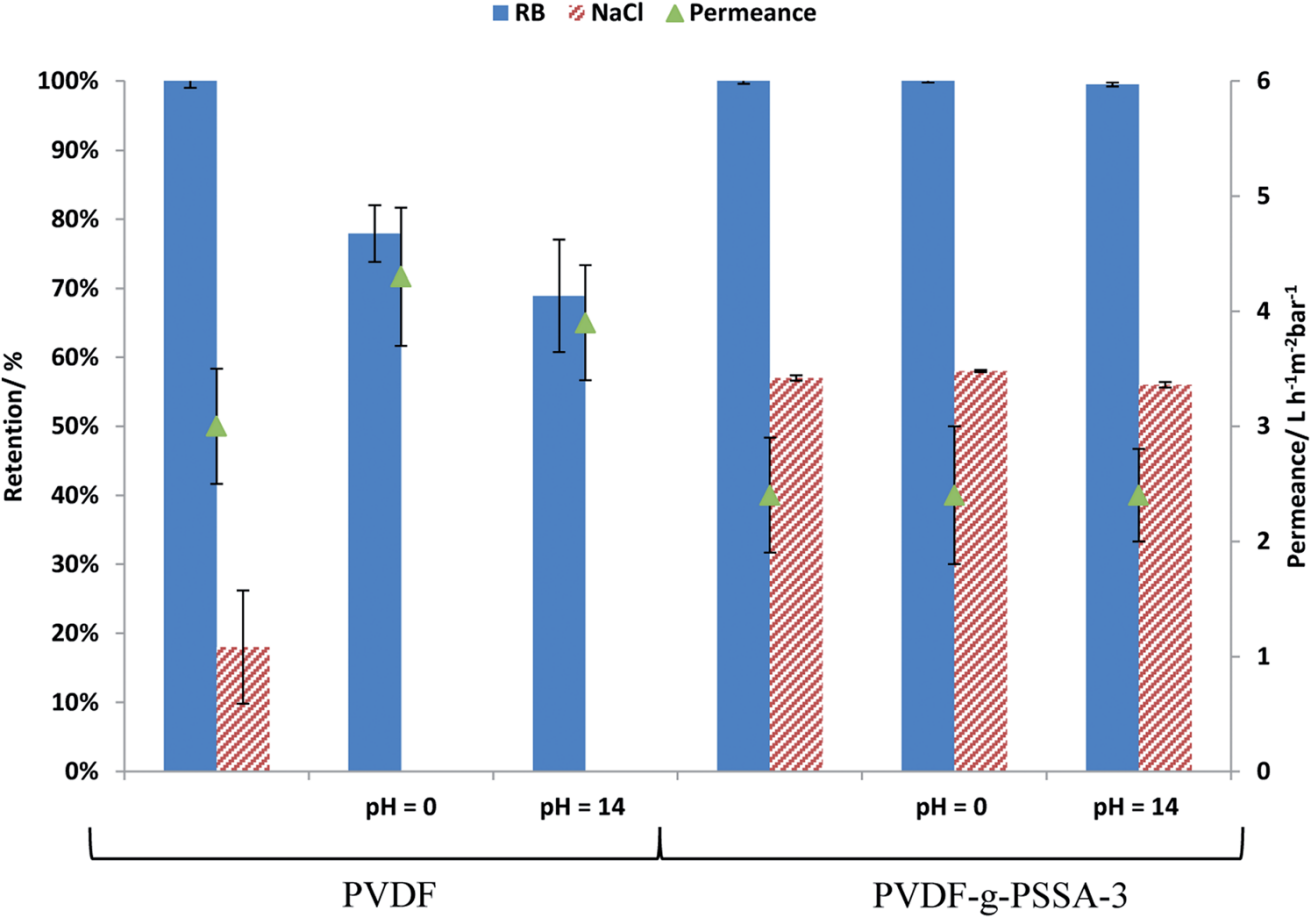
Influence of treatment at extreme pH on the permeance and on the salt and dye retention for PVDF and PVDF-*g*-PSSA-3.

PVDF membranes are not stable under alkaline conditions as a consequence of the dehydrofluorination reaction, which generates carbon–carbon double and triple bonds with the elimination of HF molecules.^28^ This process deteriorates the membrane structure by generating a more open structure with wider and larger pores.^28^ This deterioration was also observed experimentally in this work by a decline in the salt and dye retention and an increase in the permeance for the pristine PVDF membrane after exposure to alkaline conditions (1 M NaOH solution, Fig. 13). From Fig. 13, it can be observed that the retentions of both RB and NaCl decrease for the pristine PVDF membrane upon soaking for 1 week at extreme pH of 0 or 14, while it remains constant for the PVDF-*g*-PSSA-3 after such treatments. This means that the sulfonated polystyrene protects the PVDF backbone from deterioration in the presence of acid or base even during such long exposure and/or that the dehydrofluorination had reached completion already during the membrane preparation. The permeance of PVDF-*g*-PSSA-3 remained constant around 2.4 L h^−1^ m^−2^ bar^−1^ after the pH-treatments, while the permeance for PVDF increased after acid and base treatments indicating a loss of membrane integrity. Based on the SEM images before and after the pH-treatments (see Fig. S8†) no changes in morphology could be observed for the pristine and the grafted membrane. Furthermore, only minor weight losses (0.5–3%) were discovered for PVDF-*g*-PSSA-1 to -3 after either acid or alkaline treatments, while for the pristine PVDF membrane a weight loss of 14% was determined after immersion in NaOH for 1 week, further proving its instability in alkaline environment. In acidic environment only 3% weight loss was observed for PVDF. By grafting the PVDF membrane with sulfonated polystyrene, NF-membranes that are exceptionally stable in both acidic and alkaline environment were thus obtained.

### 3.4 Comparison with existing membranes

In Table 5, the filtration performance of PVDF-*g*-PSSA-3, as well as a first indication of the molecular weight cut-off (MWCO), is compared with that of other membranes that are claimed to be stable at extreme pH (both commercial and non-commercial).

**Table 5:** Membrane properties of PVDF-*g*-PSSA-3 as compared to literature and commercial data

Membrane	Permeance [L h^−1^ m^−2^ bar^−1^]	Retention [%]	MWCO (g mol^−1^)	Max. temperature (°C)	pH range
PVDF-*g*-PSSA-3	≈2.5	55–60 (NaCl)	<500 (Rhodamine B)	120 °C	0–14
PVA-APES-1.0 (ref. 9)	0.7	50–55 (NaCl)	n.d.	n.d.	0–14
HYDRACoRe70 (ref. 9)	1.7	70 (NaCl)	380	80 °C	2–13
SelRO MPF-34 (ref. 58)	2.0	35 (NaCl)	200–300	70 °C	Up to 20% NaOH
Nadir NP030 (ref. 3 and 17)	1.7	30 (NaCl)	520	95 °C	0–14
AMS A3014 (ref. 3)	2.4	≈95% (MgSO_4_)	400	50 °C	0–12

Our PVDF-*g*-PSSA-3 membrane has a remarkable combination of beneficial properties as it combines pH-stability over the full range with the highest thermal stability and best permeance reported for this type of applications. The HYDRACoRe70 membrane has a better retention, but clearly shows a more restricted pH and thermal stability. The AMS A3014 seems slightly more selective, but also suffers from more restricted thermal and pH-stability. Since PVDF-*g*-PSSA-3 combines an unprecedented combination of salt retention, permeance, thermal and pH-stability, we believe it is a promising candidate to replace the already commercially available membranes for pH-resistant nanofiltration.

## 4. Conclusions

An improved synthesis method was developed for an already existing membrane type, uniquely combining an excellent separation performance in the NF-range with complete stability over the full (0–14) pH-range. The membranes were prepared by grafting polystyrene on dehydrofluorinated PVDF UF-membranes, followed by sulfonation of the styrene groups, thus creating a dense membrane combining Donnan exclusion and size exclusion. By means of a prolonged exposure of 1 week to extreme pH-conditions (0 or 14), it was demonstrated that these membranes are remarkably resistant towards attack by concentrated acid or base. Very promising rejections (up to 60%) of a monovalent salt as NaCl was reached, while keeping the permeance at satisfactory levels (above 2.4 L h^−1^ m^−2^ bar^−1^). If desired, even higher salt retentions are expected to be achievable by further optimization of the surface pore structure of the starting ultrafiltration membrane, by densifying the selective layer through cross-linking or by further increasing grafting degree and charge density. In the future, pH-stability measurements for longer durations (>30 days) and at higher temperatures, as well as under still harsher conditions (>1 M KOH or HCl) will be performed to further verify the pH-stability of this membrane type. In conclusion, this class of membranes opens up the possibility to use nanofiltration as separation technology in a wider range of applications, including important sectors as the food, mining and paper industry.

## Author contributions

The manuscript was written through contributions of all authors. All authors have given approval to the final version of the manuscript.

## Conflicts of interest

There are no conflicts to declare.

## Supplementary Material

RA-008-C7RA13663C-s001
