# Supplementary material for: High-performance membranes with full pH-stability
Source: RSC Adv. 2018 Feb 26;8(16):8813–27. doi: 10.1039/c7ra13663c (PMC9082047; doi:10.1039/c7ra13663c)
Supplement: RA-008-C7RA13663C-s001 [file RA-008-C7RA13663C-s001.pdf]

Electronic Supplementary Material (ESI) for RSC Advances.
This journal is © The Royal Society of Chemistry 2018

High-performance membranes with full pH-stability

Nick Daems[a], Sam Milis[a], Rhea Verbeke[a], Anthony Szymczyk[b], Paolo P. Pescarmona[a,c] and Ivo F.J. Vankelecom[a,*]

[a] Centre for Surface Chemistry and Catalysis, KU Leuven, Celestijnenlaan 200F, 3001 Heverlee, Belgium

[b] Institut des Sciences Chimiques de Rennes, CNRS, UMR 6226, Université de Rennes 1, 35042 Rennes, France

[c] Chemical Engineering Group, Engineering and Technology Institute Groningen (ENTEG), University of Groningen, Nijenborgh 4, 9749 AG Groningen, The Netherlands

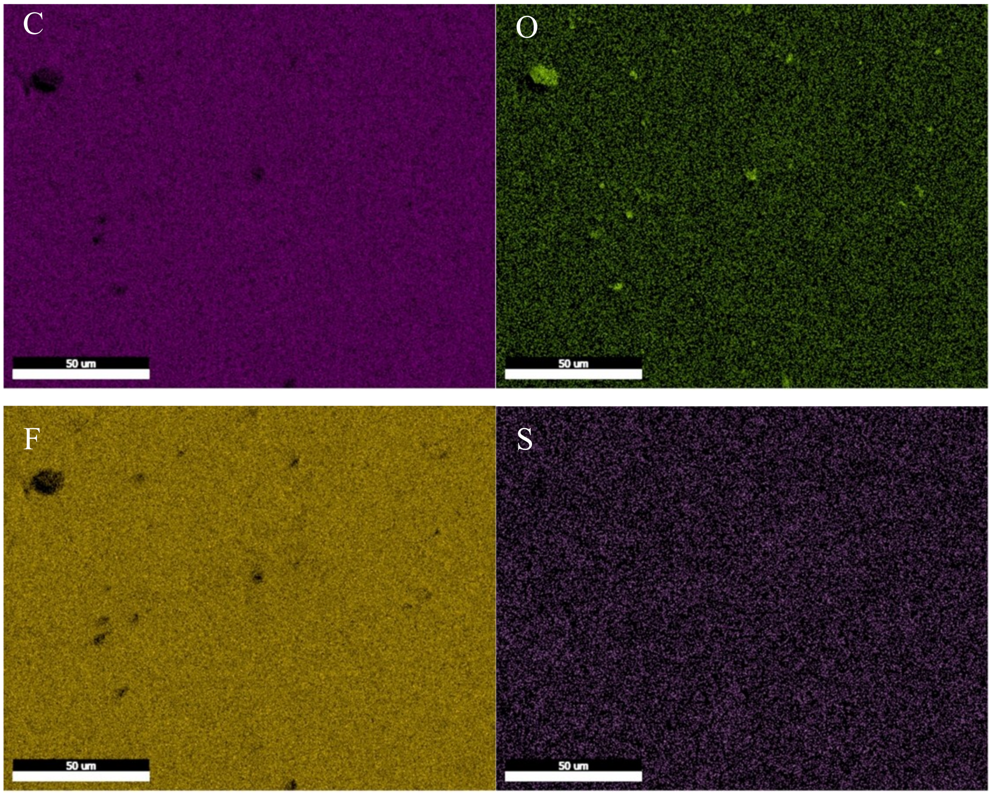

Figure S1. EDX mapping (C, O, F, S) of PVDF-g-PSSA-3 top view.

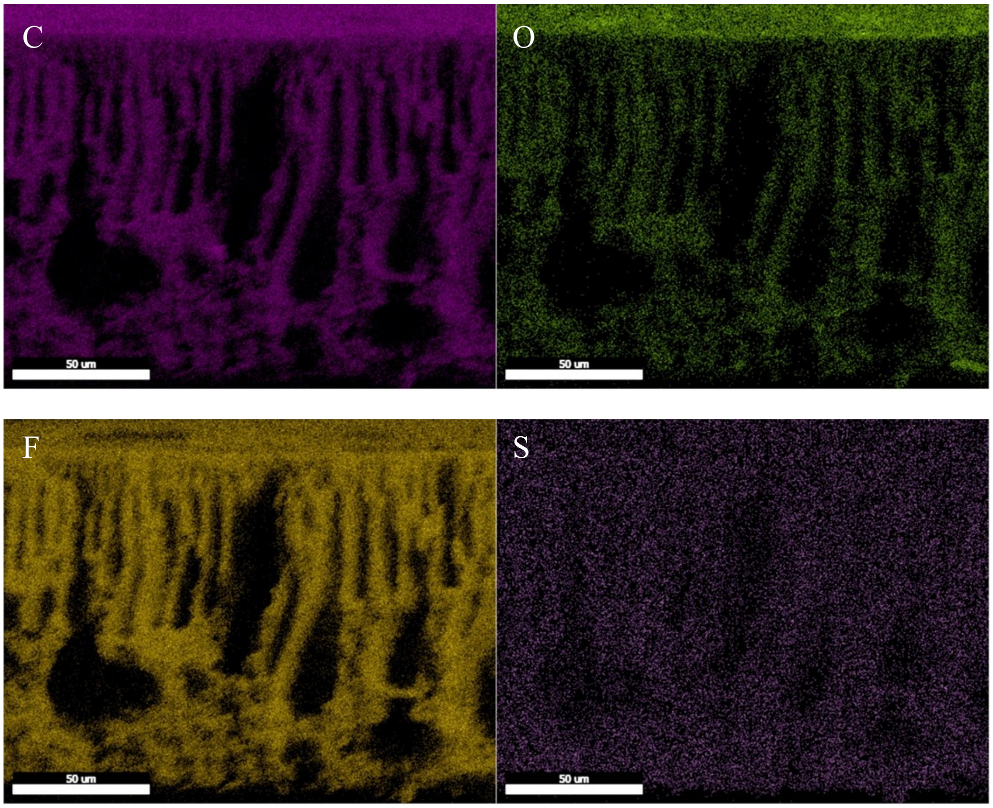

Figure S2. EDX mapping (C, O, F, S) of PVDF-g-PSSA-3 cross-section.

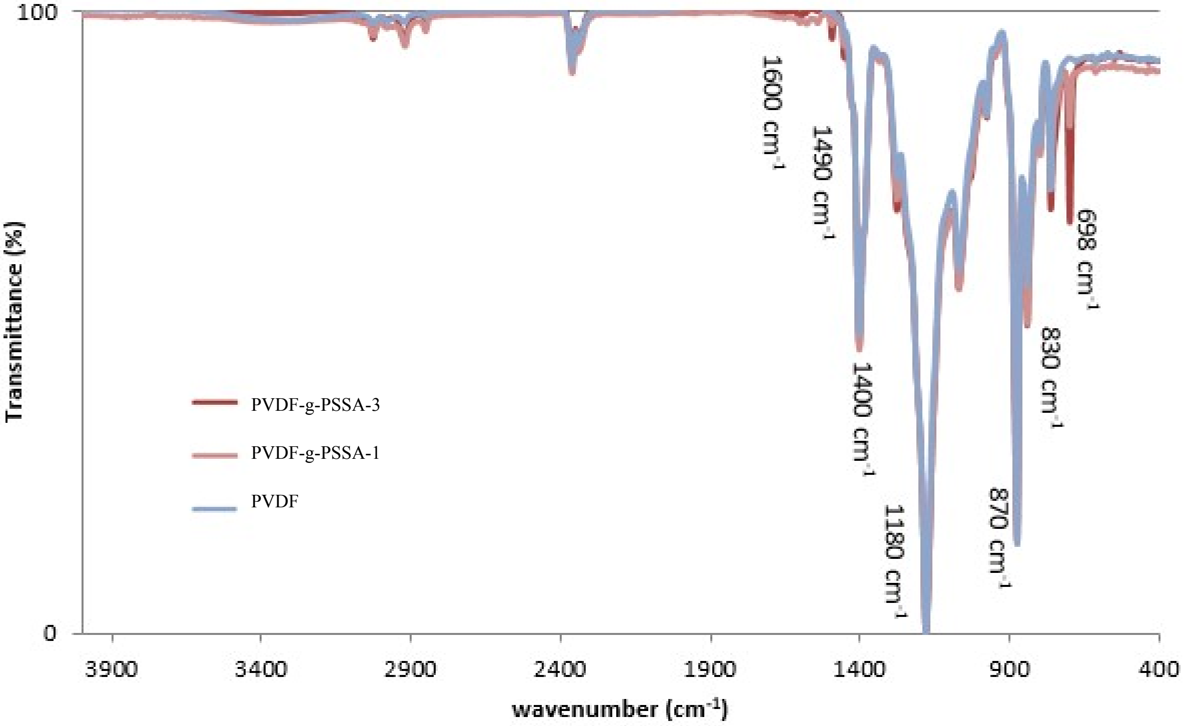

Figure S3. ATR-FTIR spectra of PVDF, PVDF-PSSA-1 and PVDF-PSSA-3 (both prior to sulfonation).

Table S1. Surface composition of different membranes as determined by XPS. Values in grey are due to sample contamination.

Sample	At% C	At% F	At% S	At% O	At% N	At% Ca	At% Na	At% Si	At% Al	At% Cl
PVDF	64.20 ± 0.23	21.46 ± 0.13	0.41 ± 0.04	9.90 ± 0.15	3.34 ± 0.18	0.24 ± 0.03	/	0.45 ± 0.13	/	/
PVDF-g-PSSA-1	62.56 ± 0.19	14.47 ± 0.12	1.09 ± 0.04	16.34 ± 0.12	3.80 ± 0.16	0.17 ± 0.03	/	0.32 ± 0.05	1.12 ± 0.08	0.19 ± 0.03
PVDF-g-PSSA-2	65.83 ± 0.18	14.87 ± 0.10	2.56 ± 0.04	13.89 ± 0.13	2.22 ± 0.16	0.22 ± 0.03	0.41 ± 0.04	/	/	/
PVDF-g-PSSA-3	64.18 ± 0.20	18.25 ± 0.10	1.87 ± 0.04	12.96 ± 0.12	2.44 ± 0.16	0.15 ± 0.03	/	/	0.16 ± 0.13	/

The origin of the different contaminating species is explained as follows. Calcium, sodium and chlorine contamination can come from the various treatments with demi-water to which the membranes were subjected. The presence of silicon can be explained by the use of a silicon grease to ensure the vacuum during the XPS measurements. The origin of aluminum remains unclear. The nitrogen presence can be attributed to residual N-methyl-2-pyrrolidone, left in the membranes from the casting solvent or from remnants of the initiator (ammoniumpersulfate). Although sulfur is also present in the pristine PVDF membrane, this value is much lower than that seen in the grafted and sulfonated samples. The sulfur 2p signal (Fig. S3) shows that there is a single sulfur environment at a binding energy of 168.2 eV, which is consistent with high oxidation state sulfur, such as sulfate or sulfonic acid, similar to that indicated for the PSSA products (Fig. S4 to S6).[1,2] Finally, the presence of oxygen in the pristine membrane can have different origins: (1) sulfate groups, (2) silicone grease, (3) NMP or (4) oxidation under air of the carbon framework.[3]

(a)

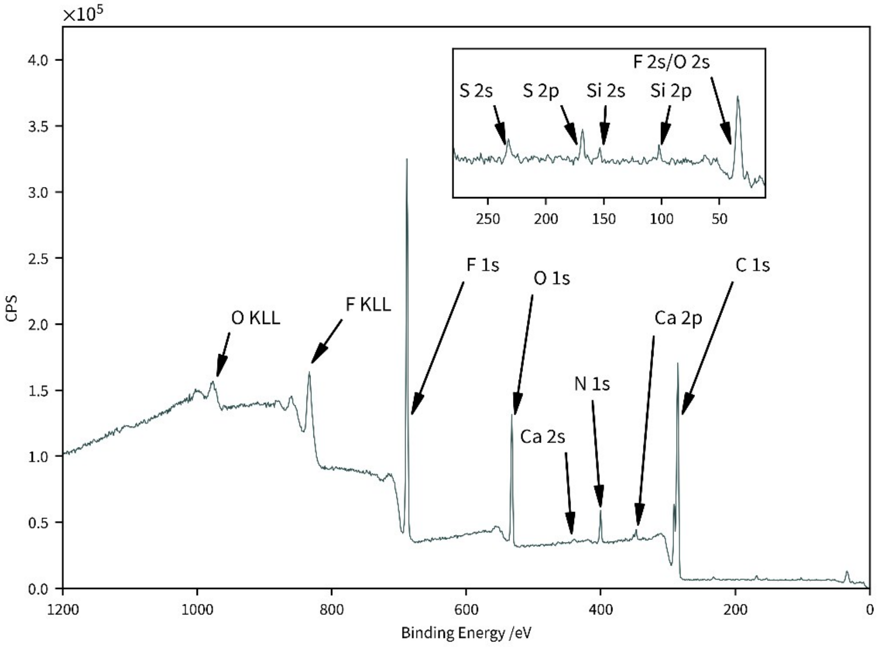

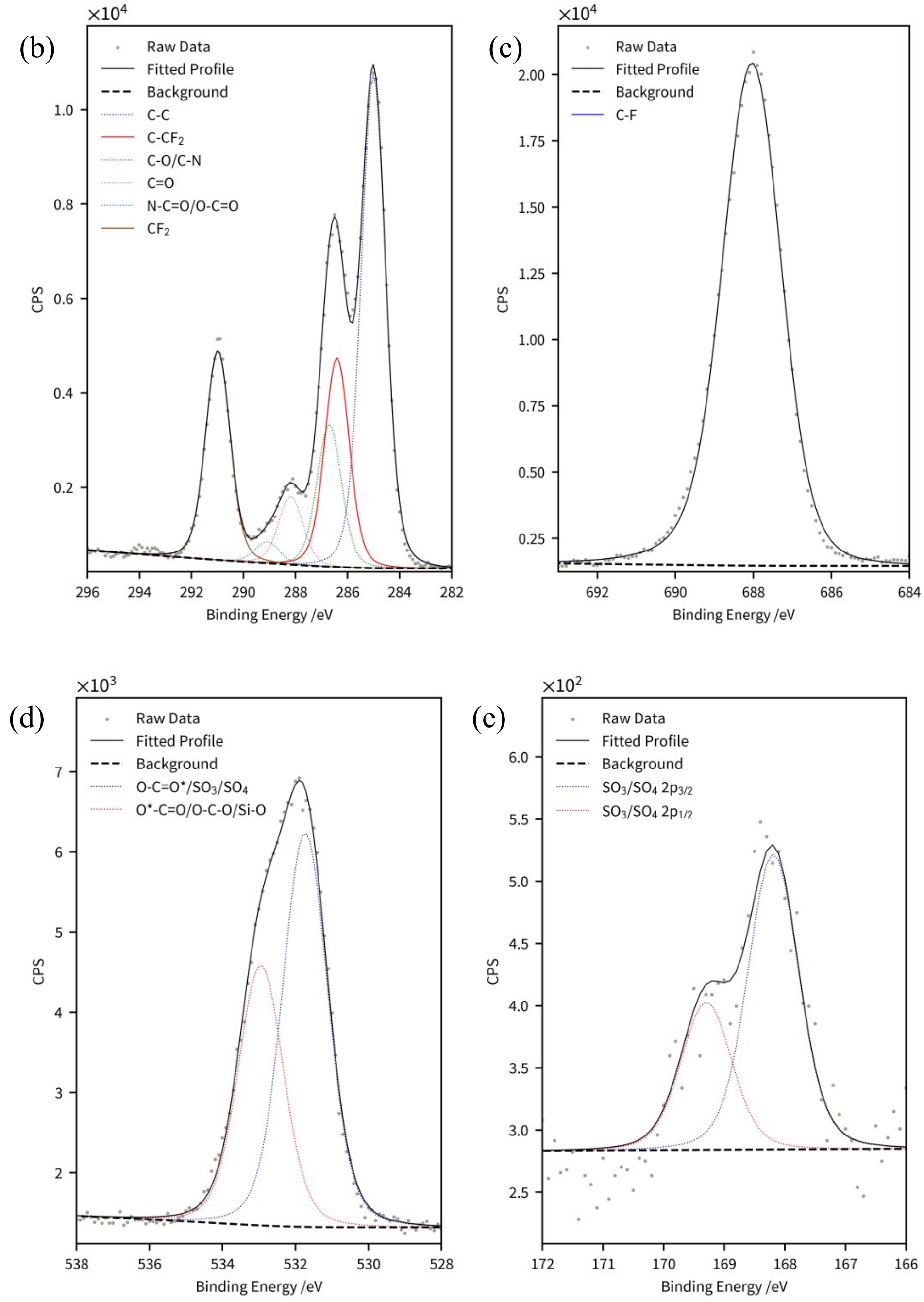

Figure S4. XPS spectra of pristine PVDF: (a) wide scan, (b) C 1s, (c) F 1s, (d) O 1s, (e) S 2p.

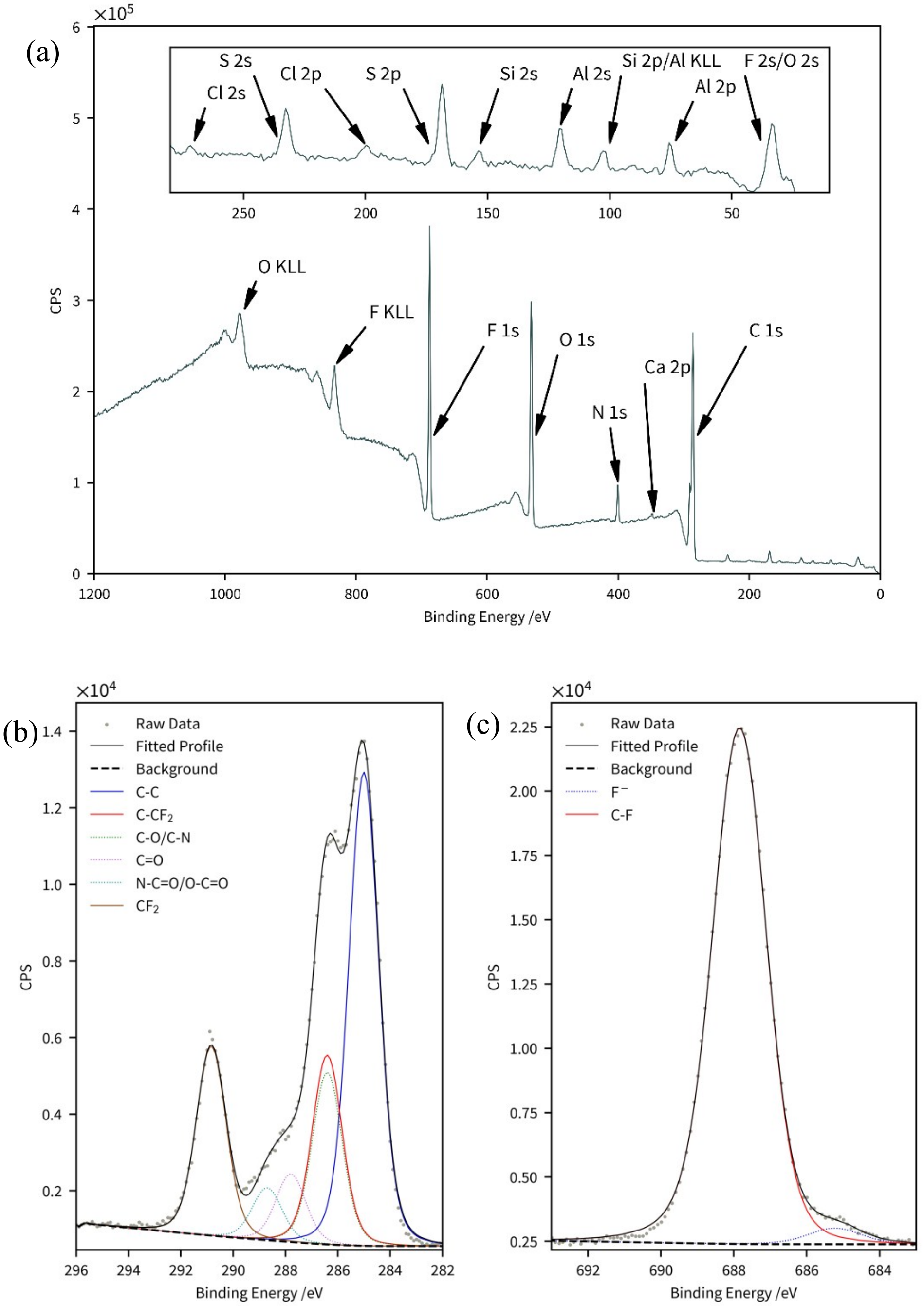
(a)
(b)
(c)
×10^5
CPS
Binding Energy /eV
O KLL
F KLL
F 1s
O 1s
N 1s
Ca 2p
C 1s
Cl 2s
S 2s
Cl 2p
S 2p
Si 2s
Al 2s
Si 2p/Al KLL
Al 2p
F 2s/O 2s
×10^4
Raw Data
Fitted Profile
Background
C-C
C-CF2
C-O/C-N
C=O
N-C=O/O-C=O
CF2
F⁻
C-F

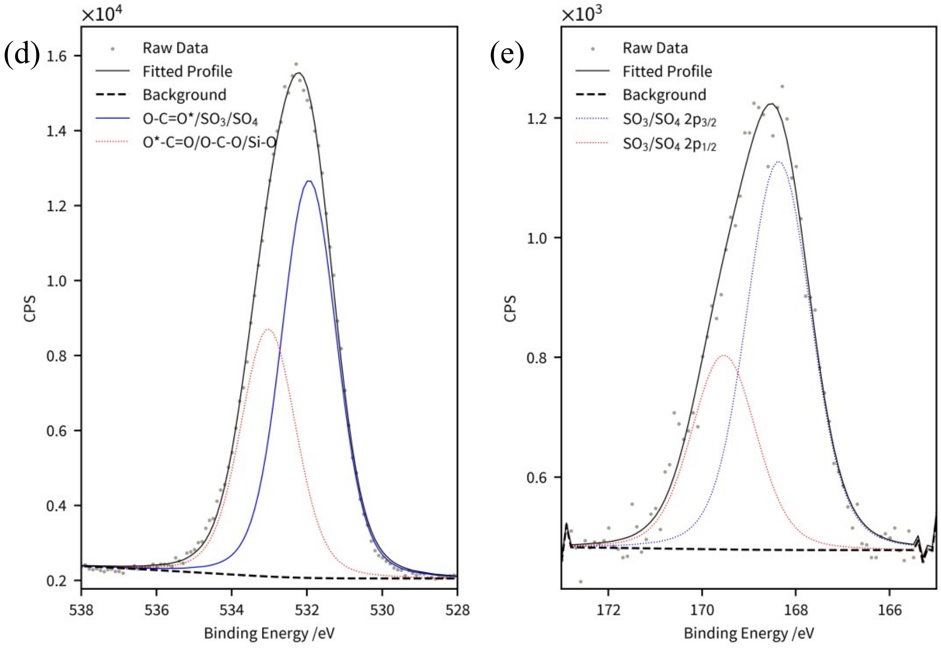

Figure S5. XPS spectra of pristine PVDF-g-PSSA-1: (a) wide scan, (b) C 1s, (c) F 1s, (d) O 1s, (e) S 2p.

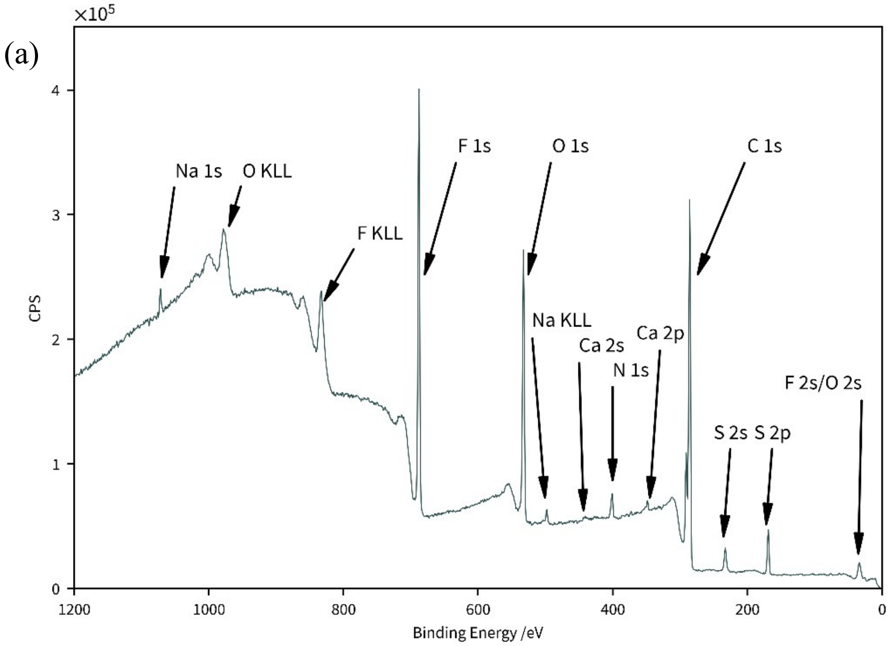

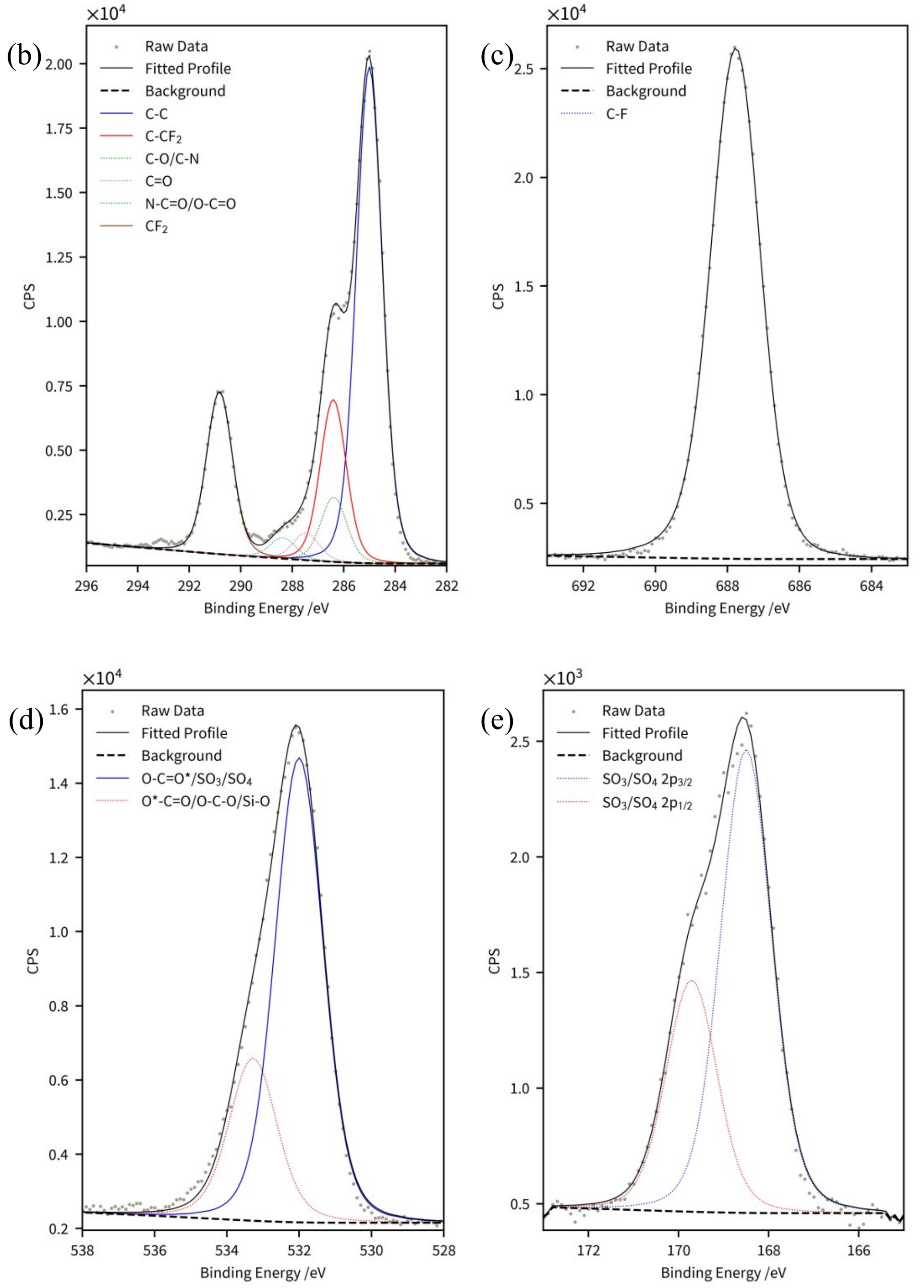

Figure S6. XPS spectra of pristine PVDF-g-PSSA-2: (a) wide scan, (b) C 1s, (c) F 1s, (d) O 1s, (e) S 2p.

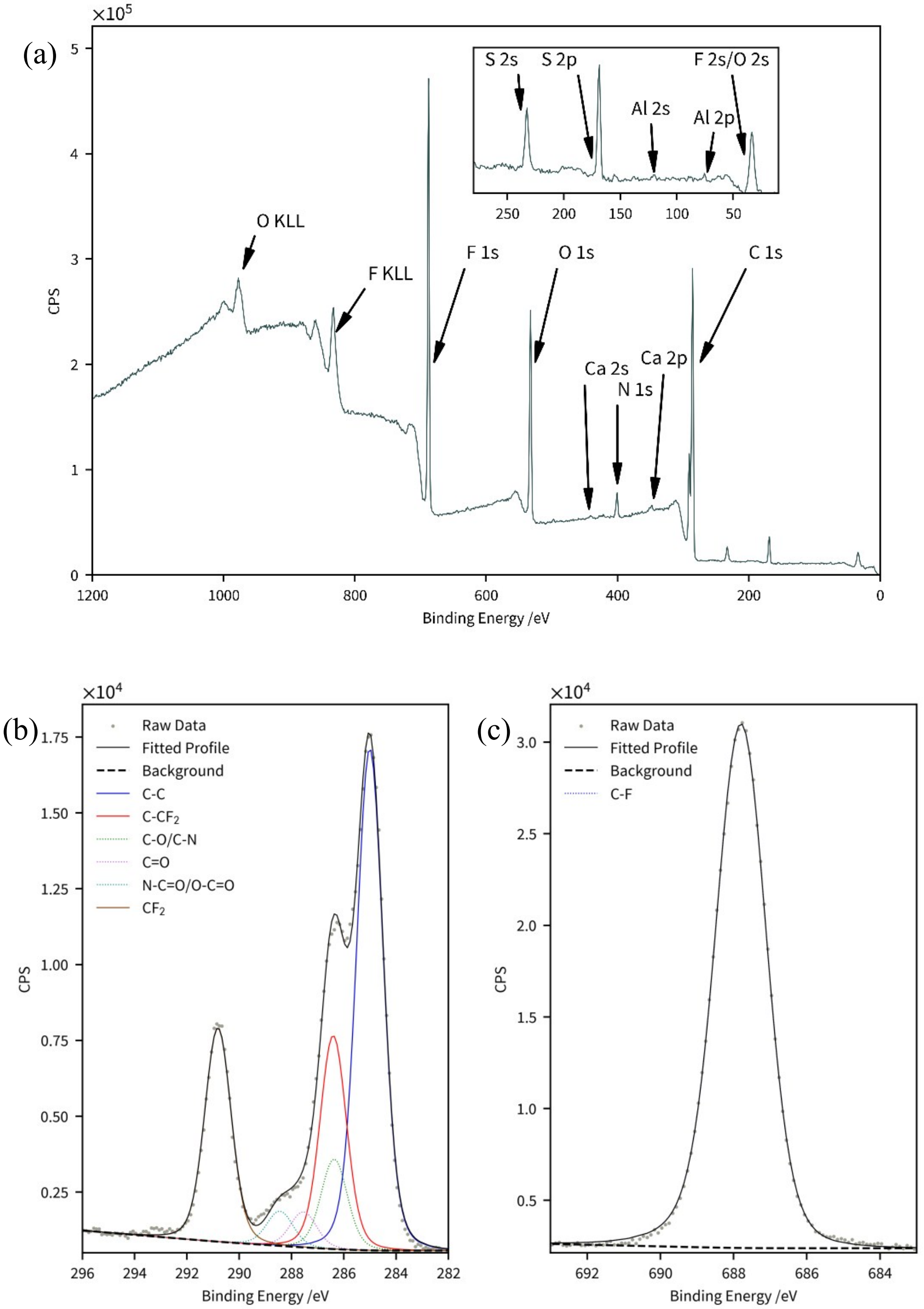

(a)
×10^5
S 2s
S 2p
F 2s/O 2s
Al 2s
Al 2p
O KLL
F KLL
F 1s
O 1s
C 1s
Ca 2s
Ca 2p
N 1s
CPS
Binding Energy /eV
(b)
×10^4
Raw Data
Fitted Profile
Background
C-C
C-CF2
C-O/C-N
C=O
N-C=O/O-C=O
CF2
CPS
Binding Energy /eV
(c)
×10^4
Raw Data
Fitted Profile
Background
C-F
CPS
Binding Energy /eV

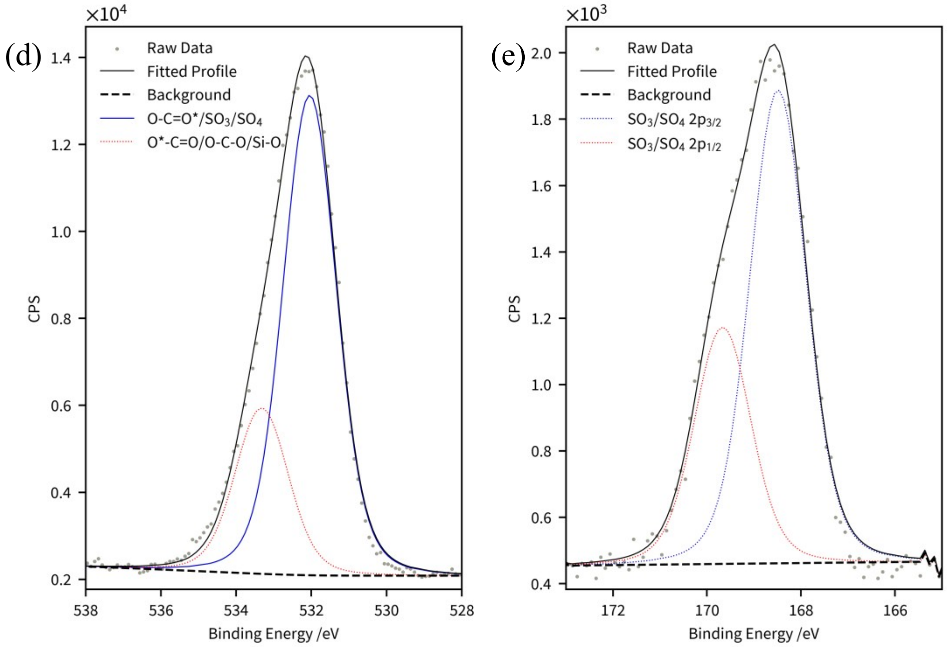

Figure S7. XPS spectra of pristine PVDF-g-PSSA-3: (a) wide scan, (b) C 1s, (c) F 1s, (d) O 1s, (e) S 2p.

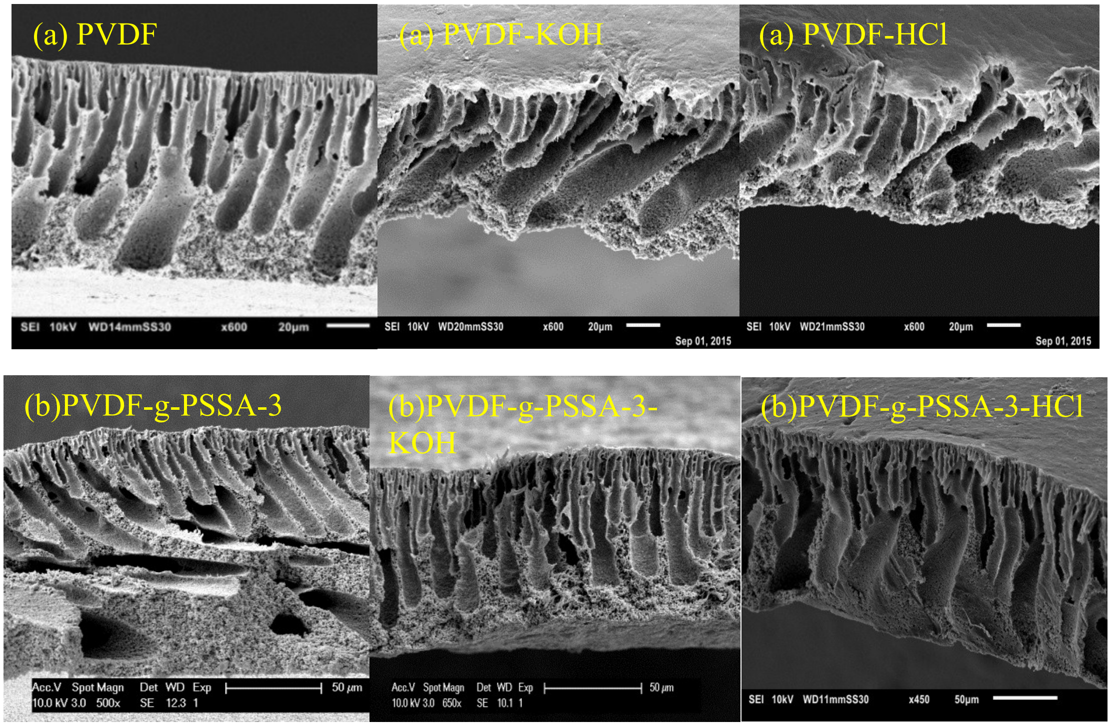

Figure S8. Cross-section SEM images before and after pH-treatment for (a) pristine PVDF membrane and (b) PVDF-g-PSSA-3 membrane.

References

1 P. Louette, F. Bodino and J.-J. Pireaux, *Surf. Sci. Spectra*, 2005, **12**, 127–132.
2 M. M. Nasef and H. Saidi, *Appl. Surf. Sci.*, 2006, **252**, 3073–3084.
3 N. Fairley, Lorentzian Asymmetric Lineshape.
